# Bioaccessibility and Transformation of Conjugated Benzotriazole Phytometabolites during In Vitro Digestion: Implications for Exposure from Recycled Irrigation Water

**DOI:** 10.1021/acs.est.5c10545

**Published:** 2025-11-18

**Authors:** Sraboni Chowdhury, Gregory H. LeFevre

**Affiliations:** † Department of Civil and Environmental Engineering, 4083 University of Iowa, 4105 Seamans Center, Iowa City, Iowa 52242, United States; ‡ IIHR—Hydroscience and Engineering, University of Iowa, 100 C. Maxwell Stanley Hydraulics Laboratory, Iowa City, Iowa 52242, United States

**Keywords:** product-to-parent reversion, emerging contaminants, phytometabolite, direct conjugation, polar trace organic contaminant, water reuse

## Abstract

Recycled water for agricultural irrigation helps to address freshwater scarcity but can contain trace levels of contaminants of emerging concern (CECs), which pose potential human exposure risks. Traditional risk assessments, focused primarily on the parent compound, overlook CECs as conjugated phytometabolites. The objective of this study was to determine the bioaccesibility and transformation of conjugated phytometabolites of benzotriazole, a prevalent micropollutant that serves as a model contaminant. *Arabidopsis thaliana* seedlings, a model Brassica plant that can be representative of other plants, including food crops, were grown hydroponically and then exposed to benzotriazole to facilitate benzotriazole uptake and phytotransformation. Plant tissues containing benzotriazole phytometabolites underwent in vitro digestion, simulating stages of human digestion (mouth, stomach, intestine). Three known phytometabolites of benzotriazole were quantified through digestion stages, and ^14^C-benzotriazole tracked mass balances. Plant-accumulated ^14^C-benzotriazole was bioaccessible with only 7% remaining in the plant tissue following in vitro digestion. The plant-accumulated mass of the individual benzotriazole phytometabolites was 2.2–3.5-fold and 4–8 times lower after gastric and intestine phases, respectively, in digested compared to nondigested plants. Gastric and intestinal phases drive bioaccessibility. High resolution mass spectrometry analysis revealed 10 novel digestion phase transformation products with distinct differences in predicted pharmacokinetic and toxicity compared to the benzotriazole phytometabolites. This study enhances understanding of bioaccessibility and transformation of CEC phytometabolites with implications for recycled water.

## Graphical abstract

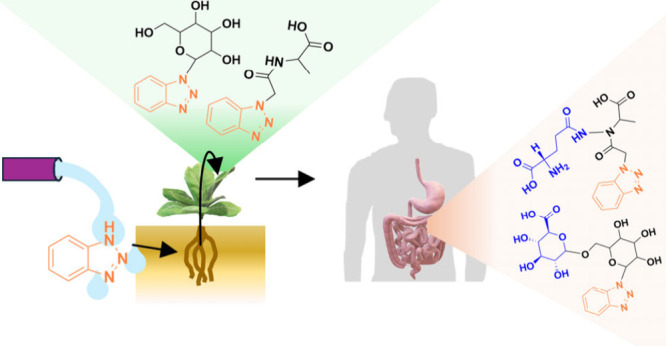

## Introduction

Use of recycled water (i.e., treated wastewater) is becoming an important alternative source of freshwater supplies for agricultural irrigation due to increased food demand from a growing population, climate change, and water scarcity.^1,2^ Many arid and semiarid regions (e.g., Middle East, U.S. Southwest) depend on recycled water to meet the high water demand for agricultural irrigation.^3,4^ Recycled water can, however, contain trace organic contaminants of emerging concern (CECs), representing a potential human exposure route when used for food crop irrigation.^5–7^ Notably, water-soluble polar CECs tend to persist in treated wastewater due to low sludge-water partitioning and limited retention time in conventional wastewater treatment processes.^8–10^ Consequently, polar CECs are more likely to enter farmland soil through recycled water used for irrigation, where high polarity and water solubility promote distribution in soil porewater and facilitate plant uptake and translocation.^5,7,11,12^ Following uptake, plants can transform and accumulate CECs as conjugated phytometabolites of unknown bioactivity that pose potential human exposure risks, despite low residual concentrations of the parent CEC. In planta transformation of CECs is classically explained via the ‘green-liver’ model of detoxification wherein compounds are functionalized (Phase I; addition of polar functional groups, e.g., −OH, −COOH, −NH_2_, −SH); conjugated (Phase II) with natural plant molecules such as glucose, amino acids, glutathione; and then irreversibly sequestered (Phase III) in cell vacuoles.^13,14^ Phase II conjugation is a key detoxification step for CECs in plants, largely driven by the presence of reactive functional groups on the CEC.^15–19^ CECs that contain certain functional groups (e.g., −OH, −NH_2_) can bypass the rate-limiting Phase I functionalization step and undergo rapid uptake by plants (e.g., ∼1 log d^–1^ in hydroponic medium for benzotriazole).^17^ CECs containing polar functional groups often undergo transporter-mediated active plant uptake, influenced by the position and electrostatic properties of the functional groups contained in the CEC.^15,20^ Presence of polar functional groups in the CEC further facilitate direct conjugation, transforming 83–99.2% of the parent compound into conjugated phytometabolites.^17,21–25^ Conjugated CEC phytometabolites can be further translocated and stored in higher plant organs.^18,26–28^ Common theoretical risk estimate approaches (e.g., Acceptable Daily Intake [ADI], Threshold of Toxicological Concern [TTC]), are based on CEC accumulation of the parent compound form only and often report minimal exposure risk^29–32^ likely because the presence of the CEC is ‘masked’ as conjugates and thus remains undetected via targeted analysis methods. Nevertheless, because the core chemical structure of the CEC within the conjugate remains unaltered and the bond connecting the conjugate to the parent CEC is typically relatively weak compared to the core CEC structure, conjugated phytometabolites could potentially revert back to the parent CEC (i.e., ‘product-to-parent reversion’) during digestion^33,34^ and yield concomitant increased consumer exposure potential.

Consumer exposure risk to CECs from recycled water irrigated food crops depends on the bioavailability of both plant-accumulated CECs and conjugated CEC phytometabolites as well as the potential transformation of CECs masked as phytometabolites during digestion. Bioaccessibility represents maximum bioavailability and is defined as the fraction of CEC released from the food matrix and available for absorption,^35,36^ which is largely unknown for CEC phytometabolites. The potential for bioaccessibility and product-to-parent CEC reversion risk during digestion with resultant release of the bioactive CECs in human gut has only recently been raised.^33,37,38^ For example, glycosylated conjugates could be hydrolyzed in the gastrointestinal tract via α-amylase or β-glycosidase enzymes capable of breaking glycosidic linkages, thereby releasing the parent aglycones from glycosylated metabolites.^39–42^ Likewise, gastrointestinal pepsin and trypsin enzymes can cleave peptide bonds adjacent to amino acids (such as alanine, arginine, lysin) and are capable of releasing the amino acid conjugated CECs.^43,44^ Such reversion to parent CEC from the glucose or amino acid conjugated metabolite increases the bioaccessible fraction of CEC in the human digestive tract.^45^ Moreover, the extent to which human metabolism can transform bioaccessible CECs or their phytometabolites is currently unknown.

Benzotriazole, a high-volume production anticorrosive with reported toxic effects including endocrine-disrupting potential, neurotoxicity, and hepatotoxicity in vertebrates,^46,47^ is frequently detected in recycled water and wastewater effluent at high concentrations (14–100,000 ng/L).^7,46,47^ Benzotriazole persists widely in aquatic environment due to high solubility, polarity, and microbial recalcitrance and demonstrates incomplete removal even by advanced wastewater treatment (e.g., RO, UV).^48^ The hydrophilicity and weak soil adsorption (*K*_d_ = 0.82 mL/g) further promote the mobility of benzotriazole through soil and ease of uptake by plants irrigated with recycled water.^7^ Our group’s previous studies demonstrated that the chemical structure of benzotriazole enables rapid active uptake by plants followed by transformation to conjugated phytometabolites through glycosylation and amino acid conjugation,^49^ similar to other small nitrogenous polar CECs.^15,20,50,51^ Benzotriazole and conjugated benzotriazole phytometabolites have also been detected in food crops irrigated with recycled water,^52^ making benzotriazole a suitable representative CEC for studying the fate of CECs from recycled water irrigated food crops to consumers. Nevertheless, key knowledge gaps remain about whether glucose and amino acid conjugated benzotriazole phytometabolites are bioaccessible under human digestive conditions, where acid hydrolysis or enzymatic transformation could release the parent CEC from the conjugated phytometabolites or transform them into other products. Therefore, the objective of this study was to determine the bioaccessibility and potential transformation of glucose and amino acid conjugated benzotriazole phytometabolites during in vitro digestion. We hypothesize that benzotriazole present in plants as undetected conjugated phytometabolites increases the exposure potential to consumers during digestion due to their bioaccessibility and potential back-transformation in the human gut. Employing multifaceted approaches such as radiolabeled benzotriazole to close mass balances, in vitro digestion with authentic standards of the benzotriazole phytometabolites, and metabolomics with high resolution mass spectrometry to discover novel products, we investigated the bioaccessibility, transformation, and novel digestion phase metabolites of benzotriazole and prevalent phytometabolites.

## Materials and Methods

### Chemicals

Full chemical details are provided in the SI (Table S1). For plant exposure, radiolabeled ^14^C-benzotriazole and unlabeled 1H-benzotriazole were used. For analytical standard preparation, glycosylated benzotriazole, benzotriazole-alanine, benzotriazole-acetyl alanine, and 1H-benzotriazole-d4 were used. All LC-MS/MS solvents used (methanol, water, and formic acid) were Optima grade. For in vitro digestion, the simulated digestion fluid was prepared following the INFOGEST 2.0 protocol^53^ (Table S2).

### Experimental Design

#### Plant Growth and Exposure to ^14^C-Labeled Benzotriazole or Unlabeled Benzotriazole

*Arabidopsis thaliana*, a widely used model plant and member of Brassicaceae family that includes many common food crops (e.g., cabbage, broccoli, radish),^54,55^ was employed for benzotriazole exposure. *Arabidopsis* seeds were sterilized using a bleach solution following our previously published procedure^15,17,20^ (detailed in the SI) and were allowed to stratify overnight at 4 °C. Sterilized seeds (*n* = 30 ± 2) were grown in sterile hydroponic Murashige and Skoog (MS) Basal medium in autoclaved Magenta GA-7-3 boxes (Bioworld) following previously published procedures.^15,17,20,56^

Following the initial growth period (11 days), plant exposure was performed as modeled in our previous work^15,17,20^ (details in the SI) in four different treatment/control groups (Figure S1). For ^14^C-benzotriazole exposed plant boxes (*n* = 30) used for the mass balance, the initial hydroponic MS medium was exchanged with ^14^C-benzotriazole (0.03 μCi/mL; equivalent to 1,665,000 total DPM). For benzotriazole exposed plant boxes (*n* = 30), medium was exchanged with sterile MS media spiked with unlabeled benzotriazole at 1.0 mg/L. This concentration is approximately 1 order magnitude higher than benzotriazole concentrations reported in treated wastewater effluent^46^ and was used for the model system to facilitate the elucidation of transformation pathways, and is recognized as higher than the environmental concentrations commonly reported in recycled water. Concurrently, one group of abiotic control boxes (*n* = 4) without plants was maintained under plant growth conditions containing ^14^C-labeled (0.03 μCi/mL or 1,665,000 total DPM) or unlabeled benzotriazole (1 mg/L) spiked MS medium only to account for any loss of benzotriazole other than plant uptake. As a negative control, one set of plant boxes (*n* = 12) was grown in MS medium only without benzotriazole exposure to account for any possible background radioactivity level or matrix effect of the experiment and ensure seed viability.

Following 5 days of BT exposure (^14^C-labeled or unlabeled benzotriazole), growth media samples (1 mL, 0.22 μm filtered) were collected and plant tissues were harvested (details in the SI) from individual Magenta boxes (working as biological replicates). Harvested plant tissues were lightly dapped dry using a Kim-wipe and added to a 15 mL centrifuge tube, and fresh weight was determined. Plant samples were frozen at −80 °C until freeze-drying. Freeze-drying was completed using a lyophilizer overnight, and dry biomass content was determined; samples were then frozen at −20 °C until in vitro digestion.

#### In Vitro Digestion Simulation

The treatment and control groups of plants for in vitro digestion included digestion treatment, negative control (digestion with plant grown without benzotriazole exposure) to account for background matrix effect, and water control (no digestion, water only exposure, benzotriazole exposed plants) to compare the effect of digestive environment (e.g., pH, enzymes, bile) (Figure S2). For the digestion treatment, benzotriazole exposed (1 mg/L unlabeled benzotriazole or 0.03 μCi/mL ^14^C-labeled benzotriazole) plants were used to simulate human digestion through three stages (*n* = 4 biological replicates of plant boxes for each phase) following the INFOGEST 2.0 protocol (Figure S3, composition details in Tables S2–S4),^53^ a static in vitro digestion model widely applied and particularly suitable for assessing the bioaccessibility of food components and phytochemicals and understanding the gastrointestinal fate of compounds.^57,58^ In brief, during the oral phase, freeze-dried plant tissues were mixed (1:1 w/v, based on fresh weight of plants) with simulated salivary fluid (pH 7.0) and α-amylase enzyme, and the mixture was stirred (150 rpm, 37 °C) for 2 min. For the gastric phase, plant digesta from the oral phase was mixed (1:1 v/v) with simulated gastric fluid (pH 3.0), gastric lipase, and pepsin enzyme and stirred (150 rpm, 37 °C) for 2 h. Following gastric digestion, the plant digesta was mixed with simulated intestinal fluid (pH 7.0), pancreatin, and bovine bile extract and stirred (150 rpm, 37 °C) for another 2 h. For the negative control samples, the three phases of digestion treatment were performed using plants grown concurrently under the same conditions but without benzotriazole exposure.

For water control samples, plants were mixed and stirred (150 rpm, 37 °C, pH 7.0 at each phase and pH 3.0 at only gastric phase) with DI water at the same dilution ratio and duration of different digestion phases to differentiate the actual contribution of the digestion system (i.e., pH, enzymes, bile) from mere water partitioning processes. Samples of the liquid digesta and digested plant tissues were sacrificially collected after each phase of simulated digestion. The liquid digesta was centrifuged at 10,000 × g for 10 min to collect the supernatant, which was then adjusted to pH 8.0. Samples of liquid digesta (1 mL, 0.22 μm filtered) were treated with enzyme inhibitors (0.1 M Pefabloc at 50 μL/mL and 10 mM orlistat at 10 μL/ml) to quench further enzymatic transformation and stored in a −80 °C freezer until analysis.

### Analytical Methods

#### Plant Tissue Extraction and Combustion for ^14^C Quantification

The digested plant tissues were extracted using 50:50 DI water:methanol following our previously established methods (details in the SI).^15,17,20^ At this stage, a group of benzotriazole (^14^C-labeled or unlabeled benzotriazole) exposed plant tissues (*n* = 4) without any digestion were extracted as a positive control to quantify the solvent-extractable fraction of ^14^C-benzotriazole and conjugated benzotriazole phytometabolites. For the plants exposed to ^14^C-benzotriazole, extracted plant tissues were freeze-dried overnight and then combusted using an OX600 Biological Oxidizer (R.J. Harvey Instruments) to quantify the unextractable/bound residue. The catalyst and combustion temperature were 680 and 900 °C, respectively. The combustion cycle lasted for 4 min. During the combustion process, ^14^CO_2_ (quantified as disintegrations per minute [DPM]) evolved from each plant residue sample was trapped (carbon dioxide traps containing 10 mL of 2 M NaOH)^59^ for subsequent analysis.

#### Radioactivity Analysis for ^14^C-Benzotriazole Mass Balance

For ^14^C-benzotriazole exposed plants, radioactivity in liquid media, liquid digesta, plant tissue extracts, and plant residue samples were quantified for 0.2 mL of liquid samples mixed with 10 mL of scintillation cocktail using liquid scintillation counting (Beckman Coulter LS 6500). To measure the background radioactivity level, liquid media, liquid digest, plant tissue extract, and plant residue samples of negative controls were used.

#### Mass Spectrometry Analysis for Quantification of Benzotriazole and Benzotriazole Phytometabolites

For unlabeled benzotriazole exposed plants, benzotriazole and benzotriazole phytometabolites (glycosylated-benzotriazole, benzotriazole-alanine, benzotriazole-acetyl alanine) in liquid media, liquid digesta, and plant tissue extract samples were quantified using high-performance liquid chromatography (Agilent 1260) coupled to a triple-quadrupole mass spectrometer (LC-MS/MS; Agilent 6460 Triple Quadrupole MS with Mass-Hunter, version B.07.00) operating in positive mode (benzotriazole, glycosylated-benzotriazole, benzotriazole-alanine) and negative mode (benzotriazole-acetyl alanine) electrospray ionization (ESI).^17,52^ The chromatography column was a Higgins Analytical Sprite Targa C18 (40 × 2.1 mm, 5 μm). The mobile phases, 0.4% formic acid in water (A) and in methanol (B), were used with variable gradients (Table S5). The MS/MS was set in multiple reaction monitoring (MRM) mode, and two MRM transition ions were used for each compound (Table S5) for quality control. An isotopically labeled *d*_4_-benzotriazole internal standard normalized external calibration curve was used to account for matrix effects during ionization for which 10 μL of 1.3 mg L^–1^ *d*_4_-benzotriazole in LCMS-grade methanol were added to each 1 mL of sample or standard. The instrument detection limits for benzotriazole, glycosylated-benzotriazole, benzotriazole-alanine, and benzotriazole-acetyl alanine were 60, 30, 36, and 34 ng/L, respectively. The signal-to-noise ratio was ≥2 for samples between nondetect and the lowest nonzero standard.

#### High-Resolution Mass Spectrometry Metabolomics for Transformation Product and Pathways Discovery

The intestine phase liquid digesta samples for digestion treatment, negative control, and water control plants were analyzed in three biological replicates (i.e., boxes of composited seedlings) using a Thermo Q-Exactive Orbitrap High Resolution Mass Spectrometer (HRMS). The digesta samples were run in full scan mode with data-dependent MS/MS (ddMS2) acquisition, using polarity switching in each sample run (i.e., both positive and negative modes were run in the same sample run). Additionally, ddMS2 scans were performed in positive and negative modes separately for composite plant tissue digesta samples (pooling 150 μL from each of three replicates) for each plant group (i.e., digestion treatment, negative control, and water control). The chromatographic and method parameters used on the Q-Exactive are detailed in the SI (Tables S6–S8).

To identify the potential digestion phase transformation products, we analyzed the HRMS data in Thermo scientific Compound Discoverer (version 3.3 SP2) using the metabolomic workflow ‘MetID w Stats Expected and Unknown with Background Removal’ customizing the workflow nodes (Figure S4). In the workflow, the targeted compounds (benzotriazole and the three benzotriazole phytometabolites) and their likely transformation pathways (Phase I and Phase II conjugation pathways detailed in the SI) were first specified. The workflow uses nodes including ‘Generate Expected Compounds’, ‘Group Expected Compounds’, and ‘Compound Class Scoring’ to predict and refine expected metabolites based on the listed targeted compounds, i.e., the parent benzotriazole or the benzotriazole phytometabolites (glycosylated-benzotriazole, benzotriazole-alanine, and benzotriazole-acetyl alanine), germane transformation pathways, ion data, and compound class information. The ‘FISh Trace’ and ‘FISh scoring’ nodes enable fragment level confirmation by matching the predicted fragment ions with experimental MS^2^ spectra. Thus, the workflow generated an ‘Expected Compounds List’ as potential transformation products of the benzotriazole and benzotriazole phytometabolites in the digesta. The Expected Compounds List was filtered to remove background with the ‘Background is false’ filter. Compounds were screened for further analysis based on a *p*-value ≤ 0.05 and the Log_2_-fold change (i.e., peak area ratio of Digestion Treatment to Negative Control or Water Control liquid digesta). Compounds with ‘Infinite’ Log_2_-fold change ratio for ‘digestion treatment/negative control’ and ‘digestion treatment/water control’ (i.e., compounds exclusively found only in the digestion treatment plant digesta) were screened. The Expected Compounds were further filtered using ‘MS2 is not equal to No MS2’, ‘FISh Coverage is greater than or equal to 5.00’, ‘Annot Mass [Da] is between −5.00 and 5.00’, and ‘Area (max.) is greater than 10^8^’. The compounds list was then sorted from the greatest to the least peak area. Candidate proposed transformation products were drawn and exported from ChemDraw 23.1.1 (PerkinElmer) to Compound Discoverer to compare the predicted fragments with the experimental MS^2^ spectra for each proposed compound applying FISh scoring. We used the Schymanski framework^60^ to communicate the confidence level of the proposed transformation products based on data in this study (Table S9).

### Computational Prediction of Pharmacokinetic and Toxicity Properties Using pkCSM

Predictions for pharmacokinetic and toxicity properties of the parent benzotriazole compound, the conjugated benzotriazole phytometabolites, and the proposed digestion phase transformation products were conducted using pkCSM, a structure-based in silico tool that uses graph-based signatures to estimate ADMET (Absorption, Distribution, Excretion, and Toxicity) properties.^61^ Canonical SMILES of each compound were generated in ChemDraw and submitted to the pkCSM web server (https://biosig.lab.uq.edu.au/pkcsm/prediction). The tool estimated hydrophilicity, intestinal absorption, volume of distribution, unbound fraction, total clearance, maximum tolerated dose, blood–brain barrier permeability, central nervous system permeability, AMES toxicity (in vitro test for mutagenicity), and hepatotoxicity. These values were used to assess and compare the absorption, distribution, excretion, and toxicity potential of the phytometabolites and novel digestion-derived transformation products of benzotriazole.

### Statistical Analysis

GraphPad Prism 9 was used for all of the statistics. A pairwise two-sided *t* test was applied to test for differences between groups or digestion phases and controls. Differences were considered significant at 95% confidence level (α = 0.05).

## Results and Discussion

### Plants Accumulate Benzotriazole As Bioaccessible Conjugated Phytometabolites

Plants assimilated and transformed benzotriazole into conjugated benzotriazole phytometabolites; some were excreted by the plants but most accumulated within plant tissue. In the benzotriazole (unlabeled) exposed plant boxes, >99.9% of the initially exposed benzotriazole was depleted from the hydroponic medium, whereas the abiotic control medium exhibited no significant (*p* = 0.9149) benzotriazole depletion compared to the initial exposure level (Figure 1a). This result indicates that benzotriazole depletion was driven by plant uptake with no notable abiotic loss, consistent with our prior work.^17^ Moreover, following the 5 days of exposure, we quantified the presence of previously reported^49,52^ glucose and amino acid conjugated benzotriazole phytometabolites in the hydroponic medium of plant control boxes (Figure 1a). This finding indicates that following initial uptake, plants transformed the parent benzotriazole into glycosylated-benzotriazole, benzotriazole-alanine, and benzotriazole-acetyl alanine then subsequently excreted these phytometabolites into the hydroponic medium, consistent with our prior findings for glycosylated-benzotriazole.^17^ Nearly one-fourth (24%) of the initially exposed benzotriazole mass (311 nanomole) was excreted as glycosylated-benzotriazole (74.4 nanomole). Excretion of glycosylated and N-glycosylated conjugates has only recently been reported; this phenomenon is likely because glycosylated metabolites are more water-soluble than the parent CEC and therefore easier to excrete from plants;^17,22,45,62^ however, whether the excretion is passive diffusion or active transporter mediated is unknown. Amino acid conjugates are known to be present as extractable residue in plants;^18,26^ however, in contrast to our previously reported results on benzotriazole phytometabolite excretion by hydroponic *Arabidopsis*,^17^ here we measured small quantities (1.2% of initial benzotriazole exposure) of excreted amino acid conjugated benzotriazole phytometabolites, benzotriazole-alanine (1.20 benzotriazole equivalent nanomole) and benzotriazole-acetyl alanine (2.54 benzotriazole equivalent nanomole). The benzotriazole-alanine and benzotriazole-acetyl alanine were also detected in bioretention cell effluent in a field tracer test with benzotriazole,^63^ demonstrating field relevance of the rapid phytotransformation of benzotriazole and excretion of benzotriazole phytometabolites by plants.

**Figure 1:**
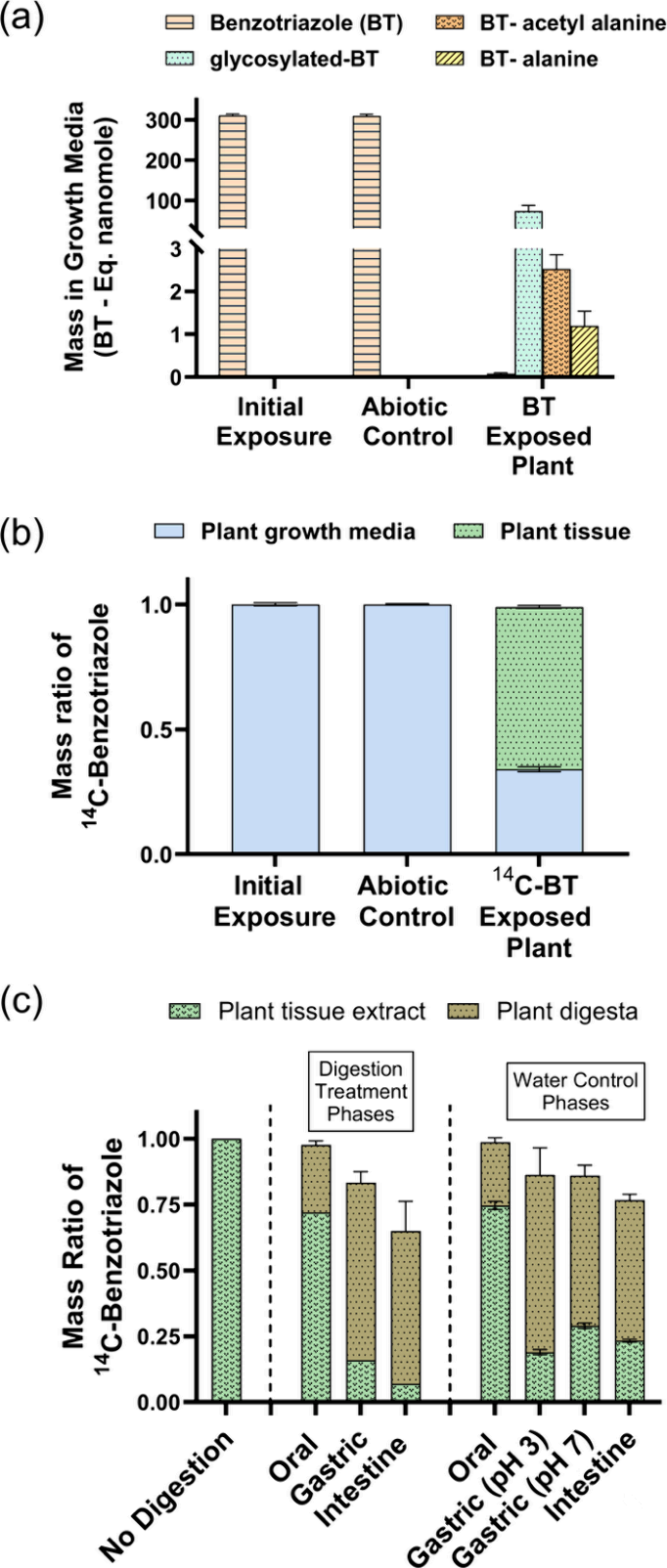
Fate and mass distribution of benzotriazole (BT) during hydroponic growth of *Arabidopsis thaliana* plants and in vitro digestion. (a) Mean mass (BT-equivalent nanomole) of BT and three conjugated BT phytometabolites (quantified with authentic standards) in hydroponic media for BT (unlabeled) exposed abiotic control boxes (*n* = 4 boxes, 30 seedlings per box) without plants and plant boxes (*n* = 30 boxes) following 5 days of exposure. (b) Mass ratio of ^14^C-BT (relative to initial ^14^C-BT) in hydroponic media of exposed abiotic control boxes (*n* = 4) and plant boxes (*n* = 30 for growth media mass and *n* = 4 for nondigested plants tissue mass). (c) Mass ratio of extractable ^14^C-BT (relative to the extractable ^14^C-BT of nondigested plant tissue) accumulated in plant tissue and released in plant digesta for digestion treatment and water control plants after each successive phase of in vitro digestion. The in vitro digestion for the digestion treatment group was performed following standardized INFOGEST 2.0 protocol (Figure S3; Tables S2–S4).^53^ For the water control groups, digestion protocol was followed (similar dilution ratio, pH, temperature, stirring, duration) using DI water only. Error bars represent the standard error about the mean.

The mass balance of ^14^C-benzotriazole (Figure 1b) provides a complementary line of evidence, illuminating plant excretion along with accumulation in plant tissue associated with exposure risks to CECs. For the abiotic control boxes, ≥99% of the initially exposed ^14^C-benzotriazole was recovered from the growth medium, indicating no appreciable abiotic losses. For the ^14^C-benzotriazoleexposed plant boxes, 34% of the initially exposed ^14^C-benzotriazole remained in the medium following 5 days of exposure (Figure 1b), which by deduction can be attributed to the plant excretion of the conjugated benzotriazole phytometabolites, as described above. The difference between the mass of ^14^C-benzotriazole and the sum of the three quantified phytometabolites (expressed as benzotriazole-equivalent molar mass) in the growth media represents the mass of excreted but unidentified benzotriazole phytometabolites because nearly no parent benzotriazole remained in the media (Figure 1a). In a similarly designed hydroponic plant uptake study of sulfamethoxazole (SMX), approximately 27% of ^14^C radioactivity remained in the media after 10 days of exposure, which was also attributed to the potential excretion of ^14^C-SMX metabolites.^28^ The fact that a substantial portion (76.5%) of the excreted benzotriazole phytometabolites can be accounted for by just three identified quantified phytometabolites—primarily glycosylated benzotriazole—demonstrates that we captured the major portion of plant excreted metabolic products. Our experimental design employed parallel ^14^C-radiolabeled isotopes and phytometabolite authentic standards and is thus able to reveal insights that would be effectively masked if using only one approach.

Despite the demonstrated plant excretion of phytometabolites described above, 65% of the initially exposed ^14^C-benzotriazole accumulated in the plant tissue prior to any digestion, with the accumulated ^14^C-benzotriazole as 10% bound residue and 55% extractable residue (Figure S5). Of the extractable residue, 78.2% could be accounted for by quantifying the three identified phytometabolites, offering further evidence that this study effectively captures the fate of dominant benzotriazole phytometabolites. Because the residues bound in plant tissue are considered less bioaccessible to the consumer compared to the extractable plant residue,^27,64,65^ we used the extractable mass prior to digestion as a reference for subsequent comparison of the bioaccessibility during the in vitro digestion of the plant tissue.

Plant accumulated ^14^C-benzotriazole mass was released from plant tissues following each phase of the in vitro digestion, demonstrating bioaccessibility (Figure 1c). Indeed, after the gastric phase, there was a significant decrease in extractable ^14^C-benzotriazole mass in the digested plant tissue (*p* < 0.001) compared to nondigested plant tissue, with only 16% of the plant-accumulated extractable ^14^C-benzotriazole (prior to digestion) remaining. A comparable decrease (19%) in plant accumulated extractable ^14^C-benzotriazole mass occurred for water control pH = 3 plants, with no significant difference (*p* = 0.9857) following gastric digestion, indicating that the acidic condition of the gastric phase primarily drives ^14^C-benzotriazole release from plant tissue. Following the intestine phase, only 7% of the extractable ^14^C-benzotriazole remained in the digested plant tissue—a significantly smaller quantity (*p* = 0.008) compared to the 23% of the extractable ^14^C-benzotriazole retained in water control plant tissue—demonstrating that the pancreatin (mixture of digestive enzymes) and bile further promoted the release of ^14^C-benzotriazole from plant tissue during intestine phase digestion.

Our observed release of ^14^C-benzotriazole from the water control plants is consistent with other in vitro digestion studies (e.g., antibiotics^37^) and may be due to the increased hydrophilicity of the phytometabolites or dissolution of water-soluble components from the plant matrix.^37^ Previous studies on in vitro digestion of *A. thaliana* report 33–55% of the initial plant biomass loss during digestion due to the dissolution of intercellular materials that comprise the plant matrix (e.g., salts and sugars in addition to the pectin material of middle lamella that contributes to ∼50% of the *A. thaliana* cell wall).^66–68^ The mass recovery of ^14^C-benzotriazole in our study for plant tissues prior to digestion was >99%, while recoveries following intestine phase digestion in the water control and in vitro condition were 81% and 77%, respectively, consistent to other in vitro digestion studies.^37^ Potential losses of ^14^C-radioactivity can occur due to the adsorption to the surface of plant tissue, freeze-drying of plant tissue, centrifugation and filtration of liquid digesta, and adsorption on tubes, filters, and vial during the sample preparation. Overall, the mass balance demonstrates that plants accumulated ^14^C-benzotriazole either in parent form or conjugated metabolite form and were released from plant tissue to the digesta, i.e., bioaccessible during in vitro digestion.

### Release and Transformation of Conjugated Benzotriazole Phytometabolites from Plant Tissue during Digestion

In vitro digestion of the unlabeled benzotriazole exposed plants revealed bioaccessibility of the glucose and amino acid conjugated benzotriazole phytometabolites from plant tissues, particularly during the gastric and intestine phase digestion. Compared to the nondigested plants, the total extractable mass of plant-accumulated benzotriazole phytometabolites (i.e., glycosylated-benzotriazole, benzotriazole-alanine, and benzotriazole-acetyl alanine) significantly decreased following the gastric phases for both the digested (*p* = 0.0001) and acidic water control (*p* = 0.0002) plants, with a 25% greater decrease in the digested treatment (*p* = 0.005) than the acidic water control (Figure 2a). For example, compared to the nondigested plants, the individual mass of extractable glycosylated-benzotriazole (μg/g dry plant) decreased by 3.5-fold in digested plants and 3-fold in the acidic water control (pH 3) plants, with a significant difference (*p* = 0.005) between the digested and acidic water control plants (Figure S6a). Moreover, the extractable benzotriazole-alanine and benzotriazole-acetyl alanine mass in the gastric phase digested plant tissue was significantly lower by 3.4-fold (*p* = 0.004) and 2.2-fold (*p* = 0.0002), respectively, compared to the nondigested plants, whereas comparing the gastric phase digested plants with water controls at pH 3, the decrease was 1.2 fold for benzotriazole-alanine (*p* = 0.0917) and 1.4-fold for benzotriazole-acetyl alanine (*p* = 0.0899) (Figure S6b,c). These findings, consistent with our observation from ^14^C-benzotriazole exposed plants, indicate that the bioaccessibility of glucose and amino acid conjugated benzotriazole phytometabolites is greatly influenced by the gastric acidic environment, which potentially promotes the breakdown and release of metabolites from plant tissue.

**Figure 2:**
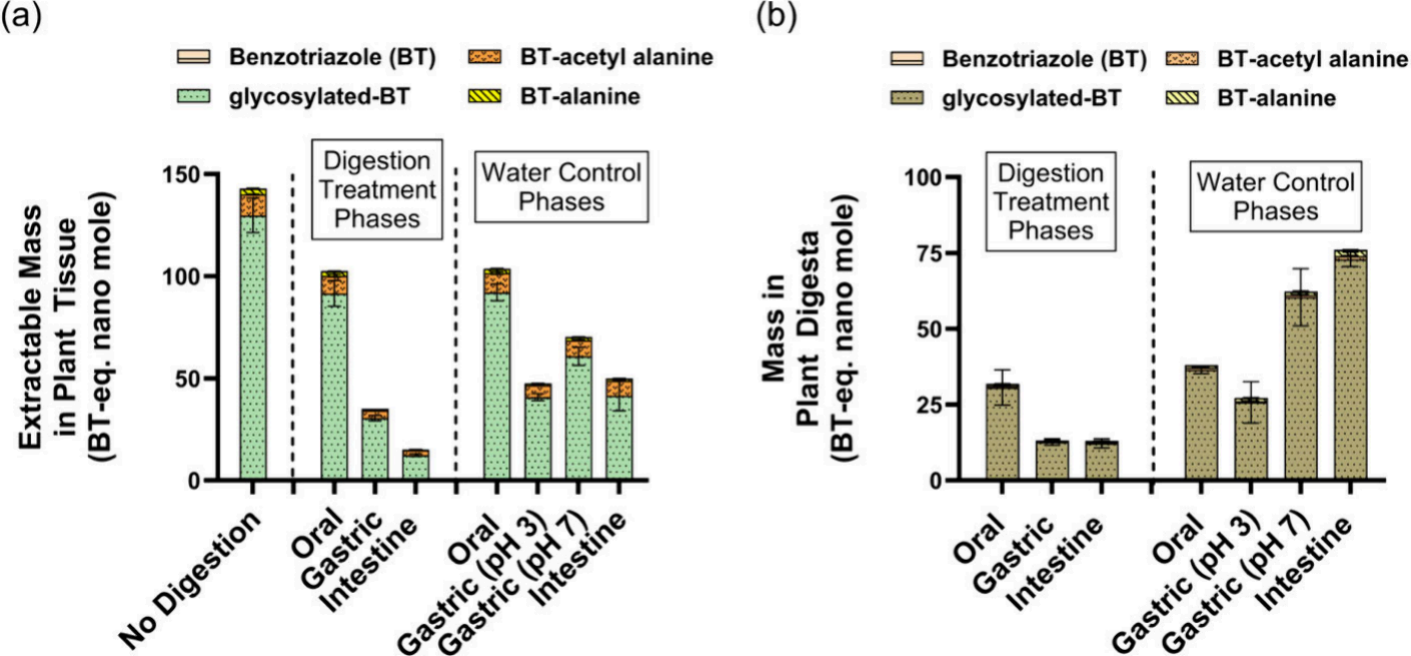
Mass distribution of benzotriazole (BT) and three conjugated BT phytometabolites following in vitro digestion. Mean mass (BT-equivalent nanomole) of BT and BT phytometabolites (measured with authentic standards) in (a) plant tissue extracted without digestion (*n* = 4 boxes; 30 seedling per box) and extracted after oral, gastric, and intestine phases for digestion treatment plants (*n* = 4 boxes for each phase) and water control plants (*n* = 4 boxes for each phase). (b) Plant tissue digesta of digestion treatment plants (*n* = 4 boxes for each phase) and water control plants (*n* = 4 boxes for each phase). In vitro digestion was performed following standardized INFOGEST 2.0 protocol (Figure S3, Tables S2–S4).^53^ For the water control groups, the same digestion protocol was followed (dilution ratio, pH, temperature, stirring, duration) using DI water only. Error bars represent standard error about the mean.

The intestinal phase led to further decreases in the total extractable masses of the benzotriazole phytometabolites in the digested plants compared to both the gastric phase digestion (*p* = 0.0069) and gastric acidic water controls (pH 3) (*p* = 0.0059), as well as the intestine water control plants (*p* = 0.0079) (Figure 2a). These results underscore the role of digestive enzymes in driving bioaccessibility during the intestine phase. In the digested plants, the extractable mass of the individual benzotriazole phytometabolites was on average 8 times (for glycosylated-benzotriazole with *p* = 0.0001 and benzotriazole-alanine with *p* = 0.0002) and 4 times (for benzotriazole-acetyl alanine with *p* = 0.0003) lower after the intestine phase compared to the nondigested plants (Figure S6). The mass of each of the individual metabolites was on average 4 times lower in the digested plants compared to the neutral water control plants, with statistically significant differences (*p* = 0.0036 for glycosylated-benzotriazole, *p* = 0.0023 for benzotriazole-acetyl alanine, and *p* = 0.0021 for benzotriazole-alanine).

The benzotriazole phytometabolites released from the plant tissue during in vitro digestion may undergo transformation within the digesta environment. Although the total mass released of the benzotriazole phytometabolites increased in the liquid digesta of water control plants following the three phases of digestion, the total mass of benzotriazole phytometabolites decreased significantly (*p* < 0.001) in the in vitro digesta samples following the gastric phase (*p* = 0.0109) and intestine phase (*p* = 0.0039) compared to the oral phase (Figure 2b). The total mass of benzotriazole phytometabolites was also significantly lower in the gastric (*p* = 0.0027) and intestinal phase (*p* < 0.0001) digesta compared to the water control (pH 7) digest. This result indicates that the released benzotriazole conjugates likely undergo transformation within the digesta environment, which aligns with topical literature. For example, an in vitro digestion study of ^14^C-sulfamethoxazole (SMX) exposed plants also reported significantly higher ^14^C-radioactivity associated with the glycosylated phytometabolite of SMX in the liquid digesta of aqueous control plants compared to the in vitro digesta; the authors suggest possible transformation of the glycosylated phytometabolite into other products during digestion due to the chemical or enzymatic condition of the digestive environment.^37^ Indeed, our observations are consistent with acid hydrolysis facilitated transformation during the gastric phase, as evidenced by a significant decrease in total mass of measured benzotriazole phytometabolites in liquid digesta of both the gastric phase digestion (*p* = 0.0027) and gastric water control (pH 3; *p* = 0.0166) plants compared to the gastric water control (pH 7) plants (Figure 2b). In the gastric phase digesta, the individual masses of glycosylated-benzotriazole and benzotriazole-acetyl alanine were significantly lower by ∼4.5-fold compared to the pH 7 water controls (*p* = 0.0027 for glycosylated-benzotriazole and *p* = 0.0006 for benzotriazole-acetyl alanine), as well as significantly lower by ∼2-fold in the pH 3 water control digesta compared to the pH 7 water control (*p* = 0.0159 for glycosylated-benzotriazole and *p* = 0.0031 for benzotriazole-acetyl alanine) (Figure S7). Potential mechanisms of transformation include acid-catalyzed hydrolysis of the N-glycosidic bond or the amide bond in benzotriazole-acetyl alanine.^69–72^ The digestive α-amylase and pepsin enzymes may have also potentially aided in transformation of the glucose and amino acid conjugated phytometabolites;^42–44,73^ indeed, the individual masses of glycosylated-benzotriazole, benzotriazole-alanine, and benzotriazole-acetyl alanine were still ∼2-fold lower in the digesta of digestion treatment plants compared to the pH 3 water control plants (*p* = 0.021 for glycosylated-benzotriazole and benzotriazole-acetyl alanine; *p* = 0.0030 for benzotriazole-alanine). Following the intestine phase, a further significant decrease (*p* < 0.0001) in the total mass of the benzotriazole phytometabolites and 3- to 6-fold decrease (*p* < 0.0001 for glycosylated-benzotriazole, *p* = 0.0014 for benzotriazole-acetyl alanine, and *p* = 0.0003 for benzotriazole-alanine) in individual mass in intestine phase liquid digesta compared to the water control pH 7 (Figure S7) provides evidence for additional transformation driven by the digestive enzymes and bile during intestine phase digestion.

Our findings are consistent with recent fate studies on phytometabolites during in vitro digestion that report acidic conditions and digestive enzymes contribute to the bioaccessibility and transformation of the conjugated CEC phytometabolites.^33,37^ Strong acidic hydrolysis in the gastric phase and pancreatin/bile in the intestine phase caused the deglycosylation of tetrabromobisphenol A mono-β-d-glucopyranoside (TBBPA MG) and TBBPA di-β-d-glucopyranoside (TBPA DG) during in vitro digestion.^33^ These studies also report that the digestive environment can revert the glycosylated phytometabolites back to the parent CEC (i.e., ‘product to parent reversion’), increasing the potential consumer exposure risk of the parent CEC. For example, the amount of parent antibiotics sulfamethoxazole and sulfamethazine released during in vitro digestion was 4–5 times higher compared to the concentrations in the plant tissues prior to digestion, which was attributed to the back-transformation of the glycosylated metabolites to the parent aglycones.^37^ In this study, we observed a steady increase in the parent benzotriazole mass in the liquid digesta of water control plants through each digestion phase, with levels in the intestine phase being 3.8 times higher (*p* = 0.0010) than those in the oral phase (Figure S7d). The increase in benzotriazole mass could result from the release of parent benzotriazole from plant tissue (Figure S6d) or the reversion of conjugated benzotriazole phytometabolites back to the parent compound. Nevertheless, when comparing the digestion-treated plants to water control plants following the intestine phase, the parent benzotriazole mass in the liquid digesta was five times lower in the digestion-treated plants (*p* = 0.0008) (Figure S7d), indicating likely transformation of the parent benzotriazole compound during in vitro digestion.

### Benzotriazole Phytometabolites Formed Novel Products during Digestion

From the results of the mass comparison of the parent benzotriazole and three different conjugated benzotriazole phytometabolites in the plant tissue extracts and liquid digesta, we observed that benzotriazole or conjugated benzotriazole phytometabolites are likely transformed into other products during digestion due to the chemical or enzymatic conditions of the digestive environment. Therefore, we employed an HRMS metabolomics-based approach with the goal of identifying the potential transformation products of the benzotriazole phytometabolites following the three-phase in vitro digestion. Samples for the HRMS metabolomics analysis were collected from the intestine phase digesta following three-phase digestion to probe the full extent of digestion transformation. Using our metabolomics approach, we were able to identify the known benzotriazole phytometabolites—glycosylated-benzotriazole, benzotriazole-alanine, benzotriazole-acetyl alanine, and benzotriazole-acetyl aspartic acid, as documented in prior work^49^—in the liquid digesta of both water control and in vitro digestion treated plants as expected transformation products of the parent benzotriazole (Confidence Level 1 except 2a for benzotriazole-acetyl aspartic acid; Table S10, Figures S9–S12). Additionally, we identified novel in vitro digestion phase transformation products resulting from Phase I activation via oxidation, hydration, oxidative deamination, or dealkylation, followed by Phase II conjugation with glucuronic acid, glutathione, and different amino acids (Figure S8). The proposed digestion phase transformation products following the phase II conjugation, i.e., conjugates (Figure 3, fully described in Table S11) are reported to Confidence Level 2b or Level 3 (Table S9, Table S11, MS2 spectra in Figures S13–S23).

**Figure 3:**
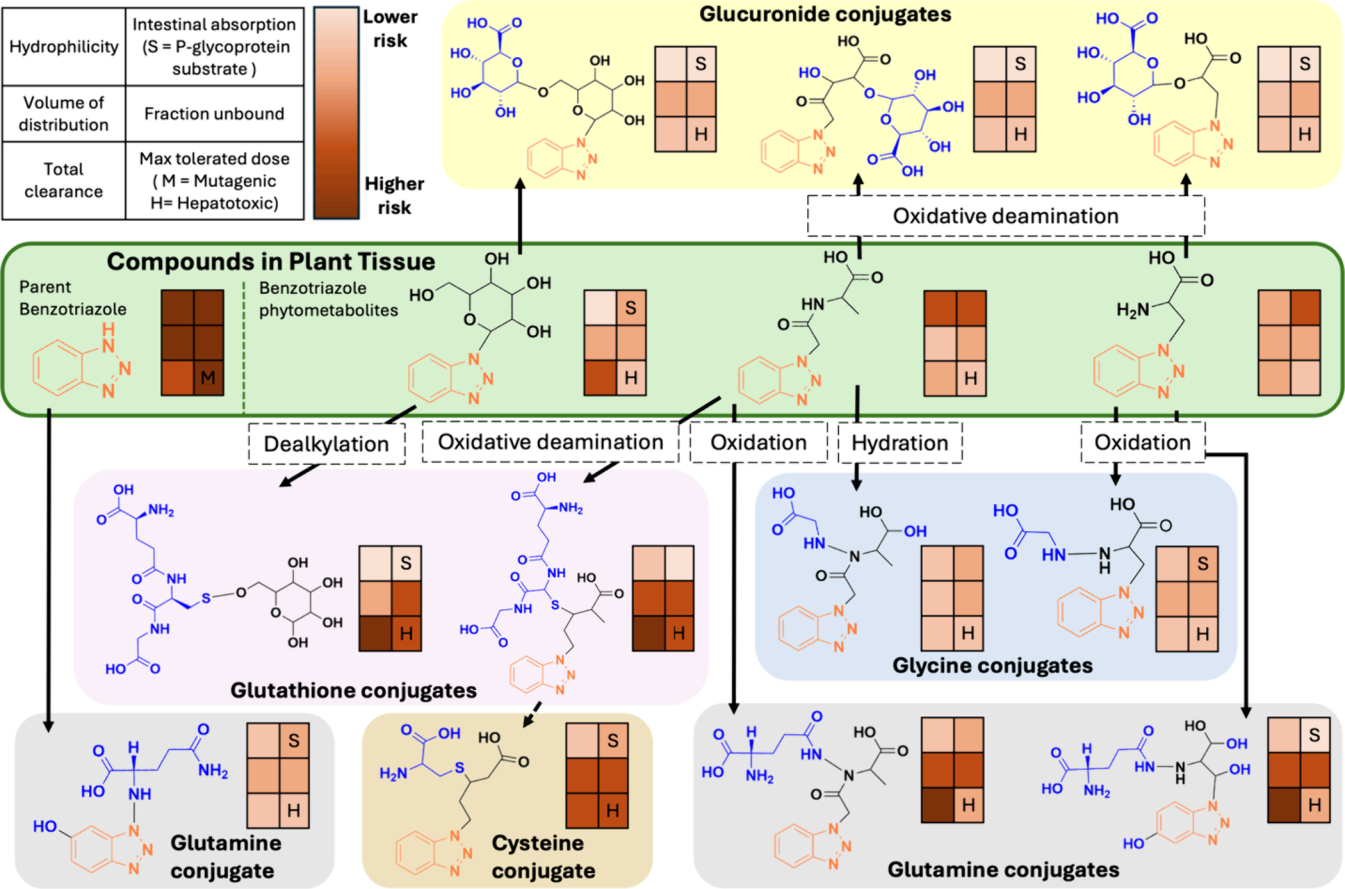
Digestion phase transformation products. Proposed transformation products of benzotriazole (BT) and BT phytometabolites observed in intestine phase digesta following the three phase in vitro digestion. The dashed line boxes represent Phase I activation processes prior to Phase II conjugation with glucuronide, glutathione, glycine, glutamine, and cysteine. Confidence levels (Schymanski framework) and annotated MS^2^ fragmentation patterns/spectra for proposed transformation products are detailed in Table S11. Note one glutathione conjugate is shown without a conjugated BT because this is how the product was discovered using the consistent postprocessing methodology; a conjugated-BT version (poor spectra) was found upon subsequent search (Figure S16). The rectangles on the right side of each structure (key in upper left of figure) represent comparative ranges of absorption, distribution, excretion, and toxicity related properties obtained using pkCSM, a predictive model for small molecule pharmacokinetic and toxicity properties.^61^ The darker shades of the rectangles signify the higher predicted percentage of human intestinal absorption, volume of distribution, and unbound fraction. The lighter the shades of the rectangles, the higher the hydrophilicity, total clearance, and max tolerated dose. The values of the properties for each compound are tabulated in Table S12.

We observed glucuronide conjugates for all three benzotriazole phytometabolites (glycosylated-benzotriazole, benzotriazole-alanine, and benzotriazole-acetyl alanine). For the benzotriazole-alanine and benzotriazole-acetyl alanine, the proposed metabolic pathway involves phase I oxidative deamination, which removes an amine group through oxidation and is often catalyzed by monoamine oxidase.^74^ Products were further metabolized via glucuronide conjugation, likely catalyzed by uridine diphosphate glucuronosyl transferases (UGTs).^74,75^ Drugs containing primary amines (e.g., Primaquine) often undergo such oxidative deamination at the terminal amine followed by glucuronide acid conjugation.^76^ Glucuronide conjugates are also reported during in vitro or in vivo (e.g., rat, zebrafish) biotransformation of benzotriazole and benzothiazoles.^77–79^ Glucuronide conjugation makes compounds more polar than the parent compound, and thus more likely to interact with membrane transporters and to cross membranes via active transport.^75^ Water-soluble glucuronide conjugates are also easier to excrete through urine or bile.^74,75^ Glucuronide conjugates may be deconjugated via gut microbiota with β-glucuronidase activity in the colon and bladder, thereby reactivating the original compound.^75,80,81^

We also observed glutathione conjugates following Phase I dealkylation and oxidative deamination for glycosylated-benzotriazole and benzotriazole-acetyl alanine, respectively. Glutathione conjugation, catalyzed by glutathione-*S*-transferase located in the liver, is a critical defense mechanism against oxidative stress that detoxifies electrophilic xenobiotic compounds/metabolites.^74^ For the glycosylated-benzotriazole, the N-dealkylation of the N-glycosidic bond caused by acidic or enzymatic hydrolysis may have detached the parent benzotriazole^41,42,82^ and formed the glutathione conjugated transformation product of glycosylated-benzotriazole (Figure 3, Figure S16). Additionally, conjugates of glutathione, a tripeptide composed of three amino acids (glycine, glutamine, cysteine), may undergo sequential enzymatic hydrolysis involving the cleavage of the glutamyl and glycine to form cysteinyl glycine, cysteine conjugates, followed by N-acetylation to produce mercapturic acids.^83–86^ The final product, N-acetylcysteine conjugates (or mercapturic), is more polar and water-soluble, thereby facilitating urinary excretion in the mammalian system.^85^ Conversion of glutathione conjugates of carbamazepine, acetaminophen, and 2-carbonitrile benzimidazole to cysteine conjugates and N-acetylcysteine conjugates have also been reported in plant metabolism studies.^15,87,88^ In this study, we observed cysteine conjugates for the benzotriazole-acetyl alanine transformation during in vitro digestion. The formation of cysteine conjugates adds complexity to human metabolic pathways through potential reactivation of the xenobiotic via β-lyase mediated reaction.^86,89^

We further discovered conjugates of the benzotriazole phytometabolites with amino acids, including glycine and glutamine, for benzotriazole-alanine and benzotriazole-acetyl alanine. Glycine conjugation, catalyzed by the glycine N-acyltransferase, is thought to be a detoxification mechanism that increases water solubility, thereby better facilitating excretion in urine.^74,90,91^ Similarly, glutamine conjugation, catalyzed by glutamine N-acyltransferase, can increase the water solubility to facilitate urinary excretion and decrease biological activity.^92^ In addition to conjugation of the benzotriazole phytometabolites, we observed glutamine conjugation of the parent benzotriazole compound. Glutamine conjugation of xenobiotics with carboxylic functional groups such as naproxen, ibuprofen, and diclofenac has also been previously observed in plant metabolism studies.^18,26,93^

The pkCSM model^61^ properties reveal distinct differences in predicted pharmacokinetics and toxicity between the parent benzotriazole, the benzotriazole phytometabolites (glycosylated-benzotriazole, benzotriazole-alanine, and benzotriazole-acetyl alanine), and the digestion phase transformation products (Figure 3, Table S12). The parent benzotriazole exhibits moderate hydrophilicity (logP = 0.96) and favorable intestinal permeability (apparent permeability coefficient, logP_app_ = 1.732), resulting in high predicted intestinal absorption (88.7%) and concomitant oral bioavailability. In contrast, benzotriazole phytometabolites are more hydrophilic (logP = 0.02 to −2.34) and have lower intestinal permeability (logP_app_ = −0.03 to −0.65), leading to lower predicted absorption (42.5–62.3%). Conjugation of the benzotriazole phytometabolites in the digestive phase markedly increases hydrophilicity and diminishes the intestinal absorption; notably, glucuronide and glutathione conjugates have 0% predicted absorption and are identified as a P-glycoprotein substrate, indicating active efflux potential.^75,94,95^ From a distribution perspective, benzotriazole phytometabolites are predicted to have a lower volume of distribution throughout the body and unbound fraction (i.e., not bound to plasma or tissue protein) compared to the parent benzotriazole. Nevertheless, the process of conjugating phytometabolites during the digestive phase with glutathione, cysteine, and glutamine increases the predicted volume of distribution and unbound fraction compared to the initial phytometabolites, indicating a potential increase in systemic bioactivity of the benzotriazole phytometabolites during digestion.^96,97^ Digestion phase glucuronide and glycine conjugates of the benzotriazole phytometabolites are predicted to enhance clearance during excretion whereas glutathione and glutamine conjugates lowers the total clearance, implying transporter-limited elimination.^98^ Toxicity prediction indicates a higher maximum tolerated dose (MTD) for the benzotriazole phytometabolites compared to the parent benzotriazole compound; however, conjugation of the benzotriazole phytometabolites in the digestion phase with glutathione, cysteine, and glutamine lowered the MTD, implying increased toxicity.^99,100^ Moreover, although benzotriazole is predicted to be mutagenic but not hepatotoxic, certain benzotriazole phytometabolites (glycosylated-benzotriazole and benzotriazole-acetyl alanine) and all digestion phase transformation products hold predicted hepatotoxicity, highlighting a shift in forecasted toxicological risk following plant uptake and in planta metabolism of the parent compound and subsequent gastrointestinal transformation of the phytometabolites. Although the conjugated phytometabolites are predicted to be less toxic compared to the parent benzotriazole—consistent with established plant detoxification mechanisms (e.g., green liver model)^13^—the phytometabolites can further transform into products with higher or lower modeled toxicity during digestion. Therefore, estimating the overall toxicological impact of CECs taken up from recycled water holds uncertainty because the accumulation of CECs as conjugated phytometabolites and the toxicity of the phytometabolites following digestion have not been specifically assessed.

### Environmental Implications

Plants can accumulate CECs as bioaccessible conjugated phytometabolites, posing an important, yet underappreciated, human exposure route through food crops. Polar CECs with reactive functional groups (e.g., −OH, −COOH, −NH_2_, −SH) can undergo rapid plant uptake, often through transporter-mediated active uptake driven by the functional group properties and position,^15,20^ and directly conjugate with endogenous plant compounds (e.g., glucose, amino acid, glutathione, sulfate) forming conjugated CEC phytometabolites.^15,18,101,102^ Direct conjugation of glucose and amino acids with CECs of similar structure/properties/reactive functional groups to benzotriazole (e.g., benzothiazole,^102^ mercaptobenzothiazole,^56^ di- or tribromophenol,^103,104^ tetrabromobisphenol,^62^ sulfamethoxazole,^50^ sulfamethazine^51^) has been previously observed, demonstrating broadly applicable plant metabolic pathways among small polar CECs and utility of benzotriazole as a model CEC.^17,21,28,62,102^ The documented accumulation of benzotriazole in crops (e.g., radish, lettuce, tomato) irrigated with recycled water following the full plant growth period (45–105 days)^7^ along with detection of conjugated benzotriazole phytometabolites (that had been previously reported in *Arabidopsis*) in roots and shoots of field grown recycled water irrigated strawberry plants,^52^ underscores the broader environmental and dietary relevance of this study. Thus, the bioaccessibility and transformation of the glucose and amino acid conjugated benzotriazole phytometabolites during in vitro digestion in this study highlights the role of ‘metabolite masking’ in underestimating the total exposure, wherein conjugated CEC metabolites evade detection in the environment because many analytical methods target only parent compounds. Nevertheless, we demonstrate that the bioaccessible conjugates hold potential to deconjugate in the gut following digestion, thereby releasing the bioactive CECs or forming new transformation products and contribute to the cumulative exposure for consumers.

Although the INFOGEST model lacks dynamic aspects (e.g., temporal variability in pH, enzyme activities) or colonic fermentation, this model is well-correlated with in vivo studies,^105,106^ making this work a valuable first step toward understanding the digestive fate of conjugated CEC phytometabolites. Indeed, the predicted pharmacokinetic and toxicity profiles of benzotriazole and conjugated benzotriazole metabolites formed through plant metabolism and subsequent digestion in consumers highlight that plant-mediated transformation and human gastrointestinal processes may alter the risk profile of the environmental emerging contaminants like benzotriazole. Therefore, future research should focus on evaluating the long-term environmental and human health impacts of the conjugates of CECs, including their potential for bioaccumulation, biotransformation, or reactivation by gut microbes and contribution to cumulative exposure risk. Furthermore, this work highlights plant excreted CEC conjugated phytometabolites as an important and overlooked environmental exposure pathway, as deconjugation in the soil root zone (e.g., deglycosylation of glycoconjugates^45^ by soil microorganism) could reintroduce CECs into the rhizosphere, representing an important future research need. This study advances the understanding of the bioaccessibility and transformation of CEC phytometabolites from crops to consumers, enabling more comprehensive dietary exposure risk assessments of CECs from recycled water irrigated food crops that are essential for improving water sustainability, protecting public health, and strengthening agricultural resilience.

## Supplementary Material



## Data Availability

High-resolution mass spectrometry data generated in this study are openly available at Iowa Research Online, DOI: 10.25820/data.007848.

## Notes

Additional materials and methods, plant growth, exposure and extraction experimental details, in vitro digestion experimental details, supplementary results, analytical methods, high-resolution MS analysis, and metabolite details (PDF)
