# Supplementary material for: Bioaccessibility and Transformation of Conjugated Benzotriazole Phytometabolites during In Vitro Digestion: Implications for Exposure from Recycled Irrigation Water
Source: Environ Sci Technol. 2025 Nov 18;59(47):25429–41. doi: 10.1021/acs.est.5c10545 (PMC12676741; doi:10.1021/acs.est.5c10545)
Supplement: Supplementary file 1 [file es5c10545_si_001.pdf]

Supporting Information

Bioaccessibility and Transformation of Conjugated Benzotriazole Phytometabolites during in Vitro Digestion: Implications for Exposure from Recycled Irrigation Water

Sraboni Chowdhury[†, ‡] and Gregory H. LeFevre[†, ‡,*]

[†] Department of Civil and Environmental Engineering, University of Iowa, 4105 Seamans Center, Iowa City, Iowa, 52242, United States

[‡] IIHR—Hydroscience and Engineering, University of Iowa, 100 C. Maxwell Stanley Hydraulics Laboratory, Iowa City, Iowa, 52242, United States

[*]Corresponding Author: gregory-lefevre@uiowa.edu; Phone: +319 335 5655, Department of Civil and Environmental Engineering, 4105 Seamans Center for Engineering, University of Iowa, Iowa City IA, 52242, United States

Total Pages (including this page): 56

Total number of Figures: 23

Total number of Tables: 12

Supporting Information Table of Contents

Additional Methods Details *S5*

Table S1: Compounds used in this work S6

Seed Sterilization Procedure **S7**

Arabidopsis Growth Procedure. **S7**

Figure S1: Different Groups during Plant Exposure S9

Plant Tissue Harvest Details **S9**

In-vitro Digestion of Plants **S10**

Figure S2: Treatment and Control Groups during In-vitro Digestion S10

Figure S3: Flow Diagram of In-vitro Digestion method following INFOGEST 2.0 [1] S10

Table S2: Composition of Simulated In-vitro Digestion Fluid S11

Table S3: Composition of Enzyme Solution for Iin-Vitro Digestion S11

Table S4: Composition of Three Phase In-vitro Digestion S12

Plant Tissue Extraction **S12**

Analytical Methods **S13**

LC-MS/MS and MRM Transition Details **S14**

Table S5: LC-MS/MS and MRM Transition Details S14

Table S6: Mobile Phase Gradient S18

Table S7: Q-Exactive Settings for Full MS Scan S18

Table S8: Q-Exactive Settings for the Positive and Negative ddMS2 .... S19

Plant Metabolomics Settings *S19*

Figure S4: Workflow tree showing the components of the Compound Discoverer automated analysis (screenshot). S19

Workflow node details: S20

Supplementary Results* *S28

Figure S5: S28

Figure S6: S29

Figure S7: S30

Figure S8: Flowchart showing the processes of parent benzotriazole transformation through plants and in-vitro digestion S31

Table S9: Description of our use of the Schymanski framework[2], including study-specific sublevels S32

Benzotriazole phytoMetabolite Details* *S33

Table S10: S33

Digestion phase transformation product Details* *S34

Table S11: S34

Table S12: pkCSM model predicted Adsorption, Distribution, Excretion and toxicity properties of the parent Benzotriazole, Benzotriazole phytometabolites and Digestion phase transformation products S37

Mass Spectra and Structures Details of Benzotriazole Phytometabolites S40

Glycosylated- Benzotriazole S40

Figure S9: MS and MS^2 spectra of Glycosylated Benzotriazole phytometabolite S40

Benzotriazole-alanine S41

Figure S10: MS and MS^2 spectra of Benzotriazole-alanine phytometabolite S41

Benzotriazole-acetyl alanine S42

Figure S11: MS and MS^2 spectra of Benzotriazole acetyl alanine phytometabolite S42

Benzotriazole-acetyl aspartic acid S43

Figure S12: MS and MS^2 spectra of Benzotriazole acetyl aspartic acid phytometabolite S43

Mass Spectra and Structures details of Digestion Phase Transformation Products S44

Products with Glucuronide Conjugation S44

Figure S13: MS and MS^2 spectra of Glucuronide conjugated Glycosylated benzotriazole.............................S44

Figure S14: MS and MS^2 spectra of Glucuronide conjugated Benzotriazole-acetyl alanine...........................S45

Figure S15: MS and MS^2 spectra of Glucuronide conjugated Benzotriazole-alanine S46

Products with Glutathione Conjugation S47

Figure S16: MS and MS^2 spectra of Glutathione conjugated transformation product of glycosylated-Benzotriazole. S48

Figure S17: MS and MS^2 spectra of Glutathione conjugated Benzotriazole-acetyl alanine............................S49

Product with Cysteine Conjugation..S50

Figure S18: MS and MS^2 spectra of Cysteine conjugated Benzotriazole acetyl alanine S50

Products with Glycine Conjugation S51

Figure S19: MS and MS^2 spectra of Glycine conjugated Benzotriazole acetyl alanine...................................S51

Figure S20: MS and MS^2 spectra of Glycine conjugated Benzotriazole alanine...S52

Products with Glutamine Conjugation S53

Figure S21: MS and MS^2 spectra of Glutamine conjugated Benzotriazole acetyl alanine S53

Figure S22: MS and MS^2 spectra of Glutamine conjugated Benzotriazole alanine...S54

Figure S23: MS and MS^2 spectra of Glutamine conjugated Benzotriazole S55

REFERRENCES...S56

ADDITIONAL METHODS DETAILS

Chemicals. For plant exposure, radiolabeled ^{14}C-Benzotriazole (ARC 4186, 55 mCi/mmol, American Radiolabeled Chemicals, 99%), and unlabeled 1H-Benzotriazole (CAS:95-14-7, Fluka Analytical, purity ≥98%) were used. For analytical standard preparation, glycosylated BT ((2R, 3R, 4S, 5S, 6R)-2-(1H-1,2,3-Benzotriazol-1-yl)-6-(Hdroxymethyl)Oxane-3,4,5-Triol,CAS:4706-40-5, Chemspace Inc, 95%), BT-alanine (2-Amino-3-(1H-1,2,3-Benzotriazol-1-yl)propanoic acid, CAS:108791-50-0, BLD Pharmatech, 95%), BT-acetyl alanine (2-(2-(1H-Benzo[d][1,2,3]Triazoll-1-yl)acetamido)propanoic acid, CAS:1807790-60-8, BLD, Pharmatech, 97%), and 1H-Benzotriazole-d4 (BT-d4; CDN isotopes, CAS:1185072-03-0) were used. For radioactivity analysis, Biosafe-II biodegradable scintillation cocktail (Research Products International Corp.) was used. Chemical properties are provided in **Table S1**. All LC-MS/MS solvents used (methanol, water, and formic acid) were Fisher Optima LC/MS grade.

For in-vitro digestion, Human salivary α-amylase (CAS:9000-90-2, Sigma-Aldrich,173.9 U/mg), Porcine pepsin (CAS: 9001-75-6, Sigma-Aldrich, ≥3200 U/mg), Rabbit gastric extract for gastric lipase (Lipolytech, >15U/mg), Bovine bile (CAS: 8008-63-7,Sigma-Aldrich,0.667 mmol/g), and Porcine pancreatin (CAS:8049-47-6,Sigma-Aldrich, 8U/mg) were used. Pefabloc (CAS:30827-99-7, Sigma-Aldrich), Orlistat (CAS:96829-58-2, Sigma Aldrich, ≥98%) were used to stop enzyme activity in stored liquid digesta sample collected following in-vitro digestion. The simulated digestion fluid was prepared following the INFOGEST® 2.0 protocol[55] with 0.5 M KCl, 0.5 M KH_2PO_4, 1M $NaHCO_3$, 2M NaCl, 0.15 M $MgCl_2(H2O)_6$, 0.5 M $(NH_4)_2CO_3$, 0.3 M $CaCl_2(H_2O)_2$, 5N NaOH and 5N HCl for pH adjustment.

Table S1: Compounds used in this work

Compound Name *(Abbreviation Used)*	***CAS Number***	***Chemical Formula***	***Molecular Weight (g/mol)***	***Structure***
**Benzotriazole [ring-14C(U)]**	Not Available	$C_6H_5N_3$	131.06	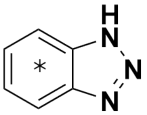
**Benzotriazole** (BT)	95-14-7	$C_6H_5N_3$	119.12	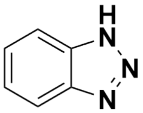
**glycosylated-Benzotriazole**	4706-40-5	$C_{12}H_{15}N_3O_5$	281.3	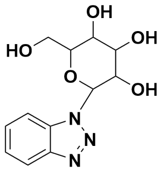
**Benzotriazole-alanine**	108791-50-0	$C_9H_{10}N_4O_2$	206.2	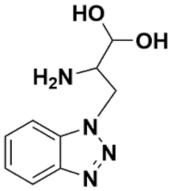
**Benzotriazole-acetyl alanine**	1807790-60-8	$C_{11}H_{12}N_4O_3$	248.24	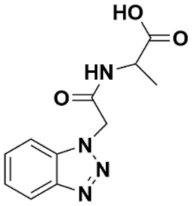
**1H-Benzotriazole-d4**	1185072-03-0	$C_6HD_4N_3$	123.15	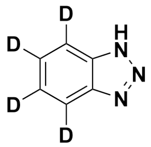

Seed Sterilization Procedure.

Wild-type *Arabidopsis thaliana* (Col-0 ecotype) seeds were sterilized using a bleach solution. In a 1.5 ml autoclaved tube 50 µL of seeds and 1 mL of seed sterilization solution were added. The seed sterilization solution consisted of 0.8 ml autoclaved DI water, 0.2 ml Bleach (8.25% sodium hypochlorite, Clorox brand) and 10 µL Tween 20 (Polyoxyethylene sorbitan monolaurate, BioRad Laboratories Inc.). The tube was vortexed briefly and slowly inverted for 5 minutes. The supernatant was removed using an autoclaved pipet. To wash the seeds from the sterilization solution, 1 mL of sterile DI water was added, vortexed briefly, slowly inverted for 5 minutes and the supernatant was removed. The washing step was repeated for a total of four times. The seeds were then stored with addition of 1 mL sterile DI water at 4°C overnight to stratify. All procedures for seed sterilization were conducted inside a biosafety cabinet. The work surface area and gloves were sterilized with 70% ethanol spray solution to create a sterile environment.

Arabidopsis Growth Procedure.

The sterilized seeds ($n = 30 \pm 2$) were added to autoclaved Magenta boxes with 25 mL of filter sterilized (0.22 µm PES, Corning) Murashige and Skoog (MS) basal medium. The MS media contained (per 1L) 4.43 g Murashige and Skoog (MS) Basal Medium powder (PhytoTech Laboratories; M519), 0.5 g 2-morpholin-4-ylethanesulfonic acid (MES) free acid monohydrate (PhytoTechnology Laboratories, CAS: 14522-94-8), 5.0 g sucrose (Research Products International) and deionized water to ~900 mL. The pH was checked and adjusted to a 5.7 as needed with 4N potassium hydroxide. Before experimental use, the medium was filter-sterilized using a bottle top filter (Corning #431118, 0.22 µm pore size) into an autoclaved bottle.

Magenta box edges were wrapped with breathable microporous tape (3M) and placed under fluorescent growth lights with a 16 h light (26 °C) and 8 h dark (22 °C) period. Plant seedlings were grown for 11 days prior to any BT exposure and were visually checked for any signs of microbial contamination. Any contaminated or poorly germinating plant boxes were discarded and not included for use in the experiments. All boxes were treated identically as biological replicates prior to BT exposure.

Plant Exposure Experiment Details

Following the 11 day period of growth in unspiked sterile hydroponic medium, the boxes were taken from the growth chamber into a biological safety cabinet and the following procedures conducted using sterile technique.

A master mix of medium was spiked with the Benzotriazole or ^{14}C-Benzotriazole. To calculate the concentration of initial exposure, 3–4 samples were taken from the master mix, at 0.5 mL each, and filtered with nylon filters (0.2 µm, 13 mm diameter, mdi SY13NN) into LC vials. These medium samples were kept frozen at -20°C until analysis.

After master mix sampling, the microporous tape was removed from each plant Magenta box and the box tilted to allow for the medium to leave the box while the plant tissue remained in the box. The box lid was then removed, and freshly spiked plant growth medium was added to each box, at 25 mL per box. The box lid and microporous tape were then replaced.

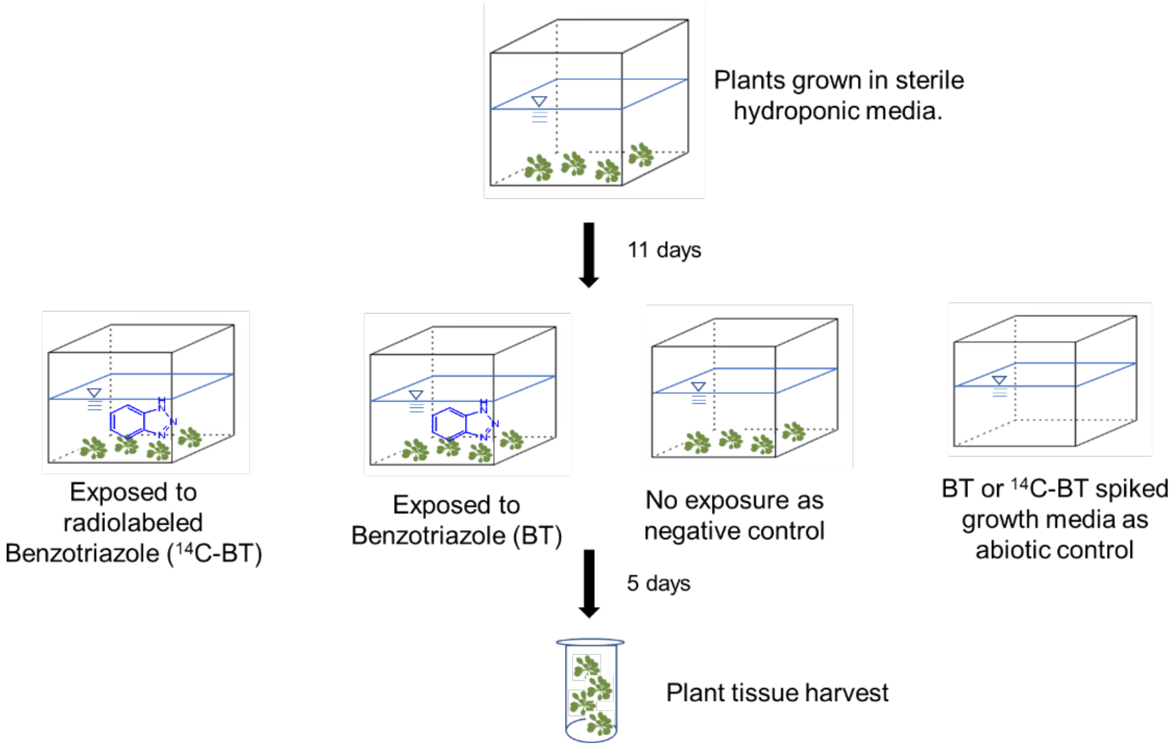

Figure S1: Different Groups during Plant Exposure

Plant Tissue Harvest Details

The microporous tape was removed from each box and the box tilted with the lid still on to allow for the medium to drain out while the plants were retained in the box. The box was then inverted into a clean paper towel, and the box removed. The plant tissue was gently dapped with the paper towel to remove any remaining medium. Clean tweezers were then used to move the tissue into 15 mL centrifuge tubes, with the tweezers cleaned with ethanol between each box.

In-vitro Digestion of Plants

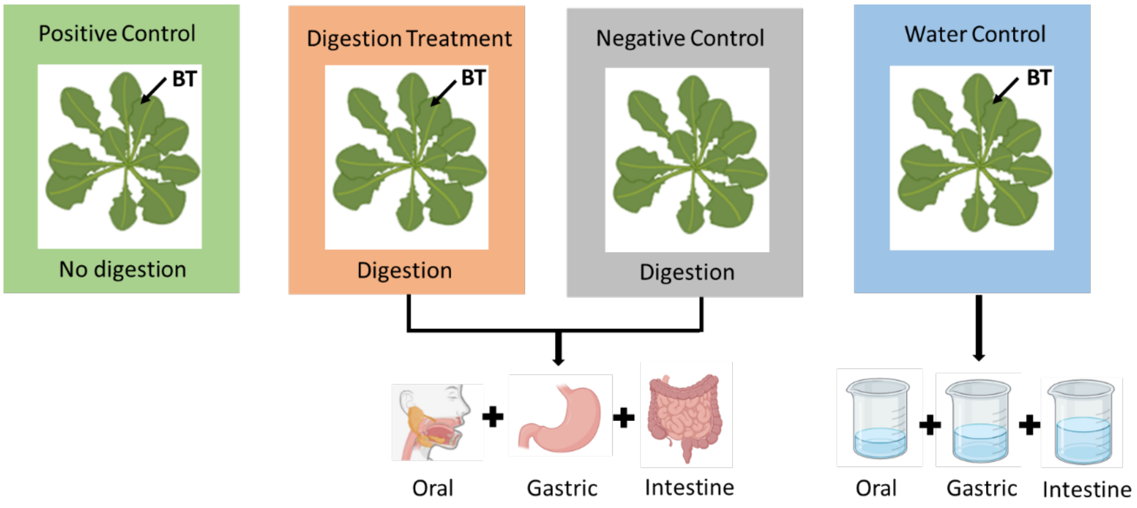

Figure S2: Treatment and Control Groups during In-vitro Digestion

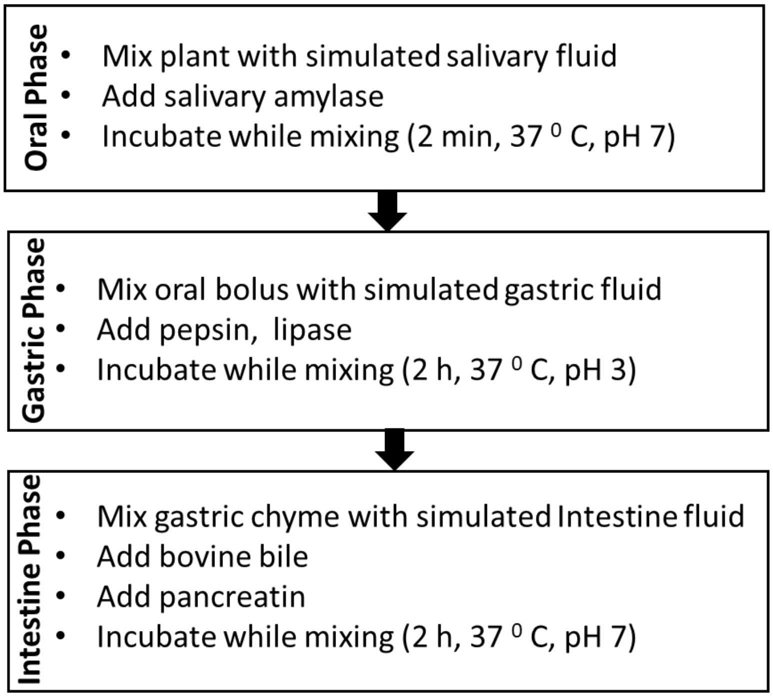

Figure S3: Flow Diagram of In-vitro Digestion method following INFOGEST 2.0 [1]

Table S2: Composition of Simulated In-vitro Digestion Fluid

Salt solution added	Stock concentration		Volume added per to prepare 0.4 L (mL)		
	(g/L)	(M)	Simulated Salivary Fluid, SSF (pH 7)	Simulated Gastric Fluid, SGF (pH 3)	Simulated Intestine Fluid, SIF (pH 7)
KCl	37.3	0.5	15.1	6.9	6.8
KH_2PO_4	68	0.5	3.7	0.9	0.8
$NaHCO_3$	84	1	6.8	12.5	42.5
NaCl	117	2	-	11.8	9.6
$MgCl_2(H_2O)_6$	30.5	0.15	0.5	0.4	1.1
$(NH_4)_2CO_3$	48	0.5	0.06	0.5	-

Table S3: Composition of Enzyme Solution for Iin-Vitro Digestion

**Digestion phase**	**Oral**	**Gastric**		**Intestine**	
Product	Sigma amylase A 1031	Lipolytech RGE 15-1G	Sigma pepsin P6887	Sigma Pancreatin 7545	Sigma Bile B3883
Enzyme solution	Salivary amylase solution	Rabbit gastric extract solution	Pepsin	Pancreatin (protease)	Bile
Final unit required (U/mL)	75	60	2000	100	10 mM

Activity (U/mg)	100	15	3200	8	0.667 mmol/g
Dissolve in water/digestion fluid (mL)	1 (water)	0.5 (water)	0.5	1 (SIF)	1 (SIF)
Concentration (mg/mL)	10	100	20	100	200

Table S4: Composition of Three Phase In-vitro Digestion

Digestion phase / Components	Oral	Gastric	Intestine
Fresh plant tissue mass or liquid digesta	1 g	2 mL	4 mL
Simulated digestion fluid	800 mL	1600 µL	1700 µL
0.3 M $CaCl_2$	5 µL	2 µL	8 µL
Enzymes and Bile solution	150 µL	160 µL (Lipase)<br>125 µL (Pepsin)	1000 µL (Pancreatin)<br>599.7 µL (Bile)
DI Water	45 µL	114 µL	692.3 µL
Final volume	2 mL	4 mL	8 mL

Plant Tissue Extraction

A single stainless steel homogenization bead (5 mm) and 1.0 mL of 1:1 methanol/water solution were added to freeze-dried plant tissues in a microcentrifuge tube. The tubes were

frozen at -80 °C for 30 min. Samples were thawed and placed on a Retsch mixer mill for 5 min at 30 Hz. The samples were then sonicated for 10 min, vortexed for 1 min, and centrifuged at 10 000 ×*g* for 10 min. Following centrifugation, the supernatant was removed with a 22G x 1 ½ BD precision glide needles needle syringe and filtered through a 0.2 µm, 13 mm diameter PFTE filter (mdi) into an empty autosampler vial. The extraction procedure was repeated sequentially two additional times by adding only 0.5 mL (rather than 1.0 mL) of the methanol: water solution for each subsequent extraction and otherwise exactly repeating the extraction procedure (i.e., homogenization, sonication, vortex, centrifugation, filtration). All three fractions were combined in a single autosampler vial for analysis.

Analytical Methods

Samples of Plant growth media, Plant tissue extract and Plant tissue digesta were analyzed via high performance liquid chromatography (Agilent 1260) coupled to a triple quadrupole mass spectrometer (LC-MS/MS; Agilent 6460 Triple Quadrupole MS with MassHunter, version B.07.00) operating in multiple reaction monitoring (MRM) positive ionization mode. LC-MS/MS method details are given below. The sample tray was maintained at 4 °C. Peak area was quantified from chromatograms using Agilent MassHunter Qualitative Analysis software and used as a proxy for concentration.

LC-MS/MS and MRM Transition Details

Table S5: LC-MS/MS and MRM Transition Details

Target Compound(s)	Chromatography	Method Parameters	Qualitative or Quantitative Transition	Q1 *m/z*	Q3 *m/z*	Dwell time (ms)	Fragmentor voltage (V)	Collision energy (V)	Cell Accelerator Voltage (V)
Benzotriazole (BT)	0.2 mL min^{-1} method for 15 min.<br><br>Mobile phase A = Fisher Optima LC/MS Water with 0.4% Optima LC/MS grade formic acid<br><br>Mobile phase B = Fisher Optima LC/MS methanol with 0.4% Optima LC/MS grade formic acid<br><br>Gradient:<br>• 0 min: 90% A, 10% B<br>• 4.0 min: 20% A, 80% B<br>• 6.5 min: 80% A, 20% B<br>• 15.0 min: 90% A, 10% B<br><br>Approximate retention time: 7.9 min<br><br>Column used: Higgins Analytical Sprite Targa C18 (40 × 2.4 mm, 5 µm)	Injection volume: 10 µL<br>Column temperature: 50 °C<br>Gas temperature: 300 °C<br>Gas flow: 8 min^{-1}<br>Nebulizer pressure: 40 PSI<br>Sheath gas temperature: 250 °C<br>Sheath gas flow: 5 L min^{-1}<br>Positive and negative capillary voltage: each 3,500 V<br>Positive and negative nozzle voltage: each 500 V<br>Polarity for all transitions: positive	Quantitative	120.06	65.1	200	75	24	4
			Qualitative	120.06	92.0	200	75	16	4

Target Compound(s)	Chromatography	Method Parameters	Qualitative or Quantitative Transition	Q1 *m/z*	Q3 *m/z*	Dwell time (ms)	Fragmentor voltage (V)	Collision energy (V)	Cell Accelerator Voltage (V)
Glycosylated Benzotriazole	0.2 mL min^{-1} method for 15 min.<br><br>Mobile phase A = Fisher Optima LC/MS Water with 0.4% Optima LC/MS grade formic acid<br><br>Mobile phase B = Fisher Optima LC/MS methanol with 0.4% Optima LC/MS grade formic acid<br><br>Gradient:<br>• 0 min: 90% A, 10% B<br>• 4.0 min: 20% A, 80% B<br>• 6.5 min: 80% A, 20% B<br>• 15.0 min: 90% A, 10% B<br><br>Approximate retention time: 3.2 min<br><br>Column used: Higgins Analytical Sprite Targa C18 (40 × 2.4 mm, 5 µm)	Injection volume: 10 µL<br>Column temperature: 50 °C<br>Gas temperature: 300 °C<br>Gas flow: 8 min^{-1}<br>Nebulizer pressure: 40 PSI<br>Sheath gas temperature: 250 °C<br>Sheath gas flow: 5 L min^{-1}<br>Positive and negative capillary voltage: each 3,500 V<br>Positive and negative nozzle voltage: each 500 V<br>Polarity for all transitions: positive	Quantitative	282.1	120.1	200	67	8	4
			Qualitative	282.1	85	200	67	12	4

Target Compound(s)	Chromatography	Method Parameters	Qualitative or Quantitative Transition	Q1 *m/z*	Q3 *m/z*	Dwell time (ms)	Fragmentor voltage (V)	Collision energy (V)	Cell Accelerator Voltage (V)
Benzotriazole Alanine	0.2 mL min^{-1} method for 15 min.<br><br>Mobile phase A = Fisher Optima LC/MS Water with 0.4% Optima LC/MS grade formic acid<br><br>Mobile phase B = Fisher Optima LC/MS methanol with 0.4% Optima LC/MS grade formic acid<br><br>Gradient:<br>• 0 min: 90% A, 10% B<br>• 4.0 min: 20% A, 80% B<br>• 6.5 min: 80% A, 20% B<br>• 15.0 min: 90% A, 10% B<br><br>Approximate retention time: 2.3 min<br><br>Column used: Higgins Analytical Sprite Targa C18 (40 × 2.4 mm, 5 µm)	Injection volume: 10 µL<br>Column temperature: 50 °C<br>Gas temperature: 300 °C<br>Gas flow: 8 min^{-1}<br>Nebulizer pressure: 40 PSI<br>Sheath gas temperature: 250 °C<br>Sheath gas flow: 5 L min^{-1}<br>Positive and negative capillary voltage: each 3,500 V<br>Positive and negative nozzle voltage: each 500 V<br>Polarity for all transitions: positive	Quantitative	207.1	120.1	200	89	8	4
			Qualitative	207.1	88.2	200	89	12	4

Target Compound(s)	Chromatography	Method Parameters	Qualitative or Quantitative Transition	Q1 *m/z*	Q3 *m/z*	Dwell time (ms)	Fragmentor voltage (V)	Collision energy (V)	Cell Accelerator Voltage (V)
Benzotriazole Acetyl Alanine	0.6 mL min^{-1} method for 12 min.<br><br>Mobile phase A = Fisher Optima LC/MS Water with 0.4% Optima LC/MS grade formic acid<br><br>Mobile phase B = Fisher Optima LC/MS methanol with 0.4% Optima LC/MS grade formic acid<br><br>Gradient:<br>• 0 min: 90% A, 10% B<br>• 3.0 min: 5% A, 95% B<br>• 6.5 min: 80% A, 20% B<br>• 7.0 min: 90% A, 10% B<br><br>Approximate retention time: 4.5 min<br><br>Column used: Higgins Analytical Sprite Targa C18 (40 × 2.4 mm, 5 µm)	Injection volume: 60 µL<br>Column temperature: 50 °C<br>Gas temperature: 200 °C<br>Gas flow: 7 L min^{-1}<br>Nebulizer pressure: 20 PSI<br>Sheath gas temperature: 250 °C<br>Sheath gas flow: 10 L min^{-1}<br>Positive and Negative capillary voltage: 2500 V<br>Positive and negative nozzle voltage: each 200- V<br>Polarity for all transitions: negative	Quantitative	247.08	118	200	113	12	4
			Qualitative	247.08	88	200	113	20	4

Samples of Plant tissue digesta of Intestine Phase were analyzed via Thermo Q-Exactive Orbitrap High Resolution Mass Spectrometer using an Agilent XDB- C18 ZORBAX, 3.5 µm, 2.1 x 50 mm column. Mobile phases used were LCMS grade water with 0.1% Formic acid (A) and Acetonitrile with 0.1% Formic acid.

Table S6: Mobile Phase Gradient

**Time (min)**	**% A**	**% B**	**Flow (mL/min)**
0	90	10	0.400
4	80	20	
8	60	40	
10	40	60	
15	10	90	
15.2	90	10	
20	90	10	

Table S7: Q-Exactive Settings for Full MS Scan

Polarity	Positive
Resolution	70,000
AGC Target	1,000,000
Max IT	200 ms
Scan Range	70-1,000 m/z

Table S8: Q-Exactive Settings for the Positive and Negative ddMS2

Resolution	17,500
AGC target	100000
Max IT	50 ms
Loop Count	3
Isolation Window	1.0 m/z
Stepped NCE	20, 40, 60
Min AGC target	4,000
Apex trigger	Not used
Exclude Isotopes	On
Dynamic Exclusion	3.0 s

PLANT METABOLOMICS SETTINGS

Figure S4: Workflow tree showing the components of the Compound Discoverer automated analysis (screenshot).

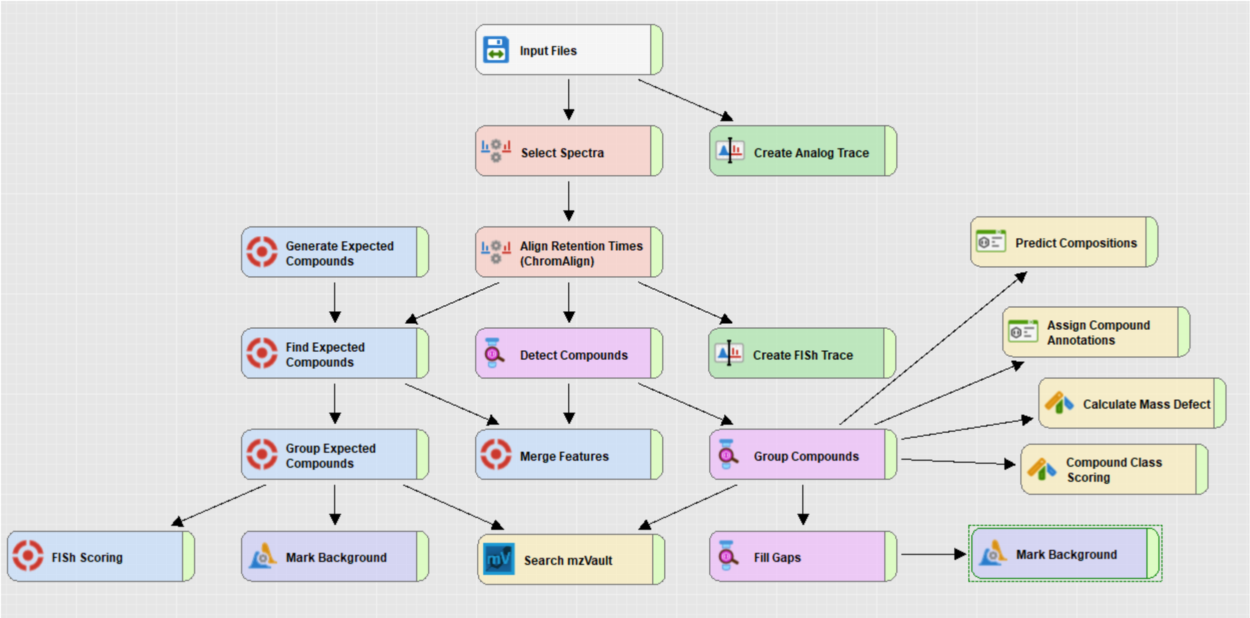

Workflow node details:

- Select Spectra
    - Lower RT Limit: 0 (if set to 0, the lowest available retention time is used)
    - Upper RT Limit: 0 (if set to 0, the highest available retention time is used)
    - Polarity mode: Any
- Align Retention Times
    - Reference file: Quality control sample file
    - Maximum Shift (min): 2
- Detect Compounds
    - Mass tolerance: 5 ppm
    - Minimum peak intensity: 1000000
    - Precursor mass tolerance: 0.025 Da
    - Ions: [2M+ACN+H]+1; [2M+ACN+Na]+1; [2M+FA-H]-1; [2M+H]+1; [2M+K]+1; [2M+Na]+1; [2M+NH4]+1; [2M-H]-1; [2M-H+HAc]-1; [M+2H]+2; [M+3H]+3; [M+ACN+2H]+2; [M+ACN+H]+1; [M+ACN+Na]+1; [M+Cl]-1; [M+DMSO+H]+1; [M+FA-H]-1; [M+H]+1; [M+H+K]+2; [M+H+MeOH]+1; [M+H+Na]+2; [M+H+NH4]+2; [M+H2O]+1; [M+H-NH3]+1; [M+K]+1; [M+Na]+1; [M+NH4]+1; [M-2H]-2; [M-2H+K]-1; [M-H]-1; [M-H+HAc]-1; [M-H+TFA]-1; [M-H-H2O]-1
- Generate Expected Compounds
    - Compound selection: Benzotriazole, Glycosylated Benzotriazole, Benzotriazole-alanine, Benzotriazole-acetyl alanine
    - Dealkylation

        - Apply dealkylation: True
        - Apply diarylation: True
        - Max# steps: 1
        - Min. Mass [Da]:150
    - Transformation
        - Phase I: Dehydration, Desaturation, Hydration, Nitro reduction, Oxidation, Oxidative deamination to alcohol, Oxidative deamination to ketone, Reduction
        - Phase II: Acetylation, Arginine conjugation, Cysteine conjugation, Glucoside conjugation, Glucuronide conjugation, Glutamine conjugation, Glycine conjugation, Glutathione conjugation, Methylation, Ornithine conjugation, Palmitoyl conjugation, Stearyl conjugation, Sulfation, Taurine conjugation
        - Max. # phase II: 1
        - Max# all steps:3
    - Ionization
        - Ions: [M+H] +1, [M+Na]+1, [M-H]-1
- Find Expected Compounds
    - General Settings
        - Mass Tolerance: 5 ppm
        - Intensity Tolerance [%]:30
        - Intensity Threshold [%]:0.1
        - Min. # Isotopes: 2

        - Use most Intense Isotope: True
        - Min. Peak Intensity: 1000000
        - Precursor Mass Tolerance: 0.025 Da
    - Peak Detention
        - Chromatographic S/N: 1.5
        - Remove baseline: False
- Group Expected Compounds
    - General Settings:
        - RT Tolerance[min]: 0.1
        - Minimum Valley [%]: 10
        - Align peaks: False
        - Preferred Ions: [M+H]+1, [M+Na]+1, [M-H]-1
        - Area Integration: Most Common Ion
    - Peak Rating Contribution:
        - Area Contribution: 3
        - CV Contribution: 10
        - FWHM to Base Contribution: 5
        - Jaggedness Contribution: 5
        - Modality Contribution: 5
        - Zig-Zag Index Contribution: 5
    - Peak Rating Filter
        - Peak Rating Threshold: 0
        - Number of Files: 0

- FISh scoring
  - General Settings
    - Annotate Full tree: True
    - Match Transformations: True
    - S/N threshold: 3
    - High Acc. Mass tolerance: 2.5 mmu
    - Low Acc. Mass tolerance: 0.5 Da
  - Fragment Prediction Settings
    - Use General Rules: True
    - Use Libraries: True
    - Max. Depth: 5
    - Aromatic Cleavage: True
    - Min. Fragment m/z: 50
- Mark Background
    - Max. Sample/Blank: 5
    - Max. Blank/Sample: 0
    - Hide Background: True
- Merge Features
  - Peak Consolidation
    - Mass Tolerance: 5 ppm
    - RT tolerance: 0.1
- Create FISh Trace
  - Compound Selection

        - Compound: Benzotriazole
    - Trace Settings
        - Mass Tolerance: 2.5 mmu
        - Summed Trace: True
        - Individual Traces: True
        - Custom Label: FISh Trace
    - Scan Filter Settings
        - Scan Polarity: +
        - Fragment Mode: Data Dependent
    - Fragment Prediction Settings
        - Use General Rules: True
        - Use Libraries: True
        - Max. Depth: 5
        - Aromatic Cleavage True
        - Min. Fragment m/z: 50
- Group Compounds
    - General Settings
        - Mass Tolerance: 5 ppm
        - RT Tolerance [min]: 0.2
        - Minimum Valley [%]: 10
        - Align Peaks: False
        - Preferred Ions: [M+H]+1; [M-H]-1
        - Area Integration: Most Common Ion

- Peak Rating Contributions:
    - Area Contribution: 3
    - CV Contribution: 10
    - FWHM to Base Contribution: 5
    - Jaggedness Contribution: 5
    - Modality Contribution: 5
    - Zig-Zag Index Contribution: 5
- Peak Rating Filter
    - Peak Rating Threshold: 0
    - Number of Files: 0

- Predict compositions
  - Prediction Settings
    - Mass Tolerance: 5 ppm
    - Min. Element Counts: C H
    - Max. Element Counts: C90 H190 Br3 Cl4 N10 O18 P3 S5
    - Min. RDBE: 0
    - Max. RDBE: 40
    - Min. H/C: 0.1
    - Max. H/C: 4
    - Max. # Candidates: 10
  - Pattern Matching
    - Intensity Tolerance [%]: 30
    - Intensity Threshold [%]: 0.1

        - S/N Threshold: 3
        - Use Dynamic Recalibration: True
    - Fragments Matching
        - Use Fragments Matching: True
        - Mass Tolerance: 5 ppm
        - S/N Threshold: 3
- Assign compound annotations
    - General Settings: Mass Tolerance: 5 ppm
    - Data Sources
        - Data Source #1: mzCloud Search
        - Data Source #2: mzVault Search
        - Data Source #3: Predicted Compositions
        - Data Source #4: Masslist Search
    - Scoring Rules
        - Use mzLogic: True
        - Use Spectral Distance: True
        - SFit Threshold: 20
        - SFit Range: 20
    - Reprocessing: Clear Names: False
- Calculate mass defect
        - Fractional mass: False
        - Standard mass defect: True
        - Relative mass defect: False

        - Kendrick mass defect: False

- Compound class scoring
    - Compound class: Benzotriazole
    - S/N threshold: 3
    - High Acc. mass tolerance: 0.5 Da
    - Low Acc. mass tolerance: 0.5 Da
    - Use full MS tree: True
    - Use DIA scoring: True
- Fill Gaps
    - Mass Tolerance: 5 ppm
    - S/N Threshold: 1.5
- Mark Background
    - Max. Sample/Blank: 5
    - Max Blank/Sample: 0
    - Hide Background: True
- Search mzVault
    - Compound Classes: All
    - Library: Autoprocessed; Reference
    - Search MSn Tree: False
    - DDA Search
        - Identity Search: Cosine
        - Match Activation Type: True
        - Match Activation Energy: Match with Tolerance

- Activation Energy Tolerance: 20
- Apply Intensity Threshold: True
- Similarity Search: Confidence Forward

SUPPLEMENTARY RESULTS

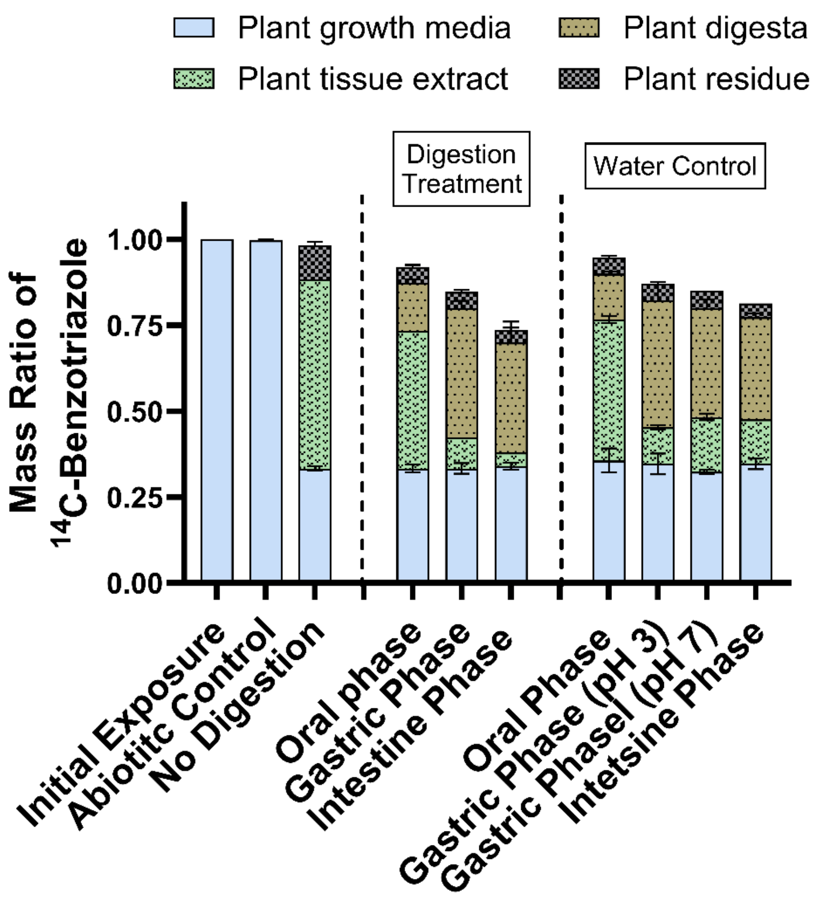

Figure S5: Mass ratio of 14C-BT (relative to the initial exposure mass of 14C-BT) in plant growth media, plant tissue extract, plant digesta and plant residue for No digestion, digestion treatment and water control plants after each successive phase of in-vitro digestion. The error bars represent the standard error about the mean.

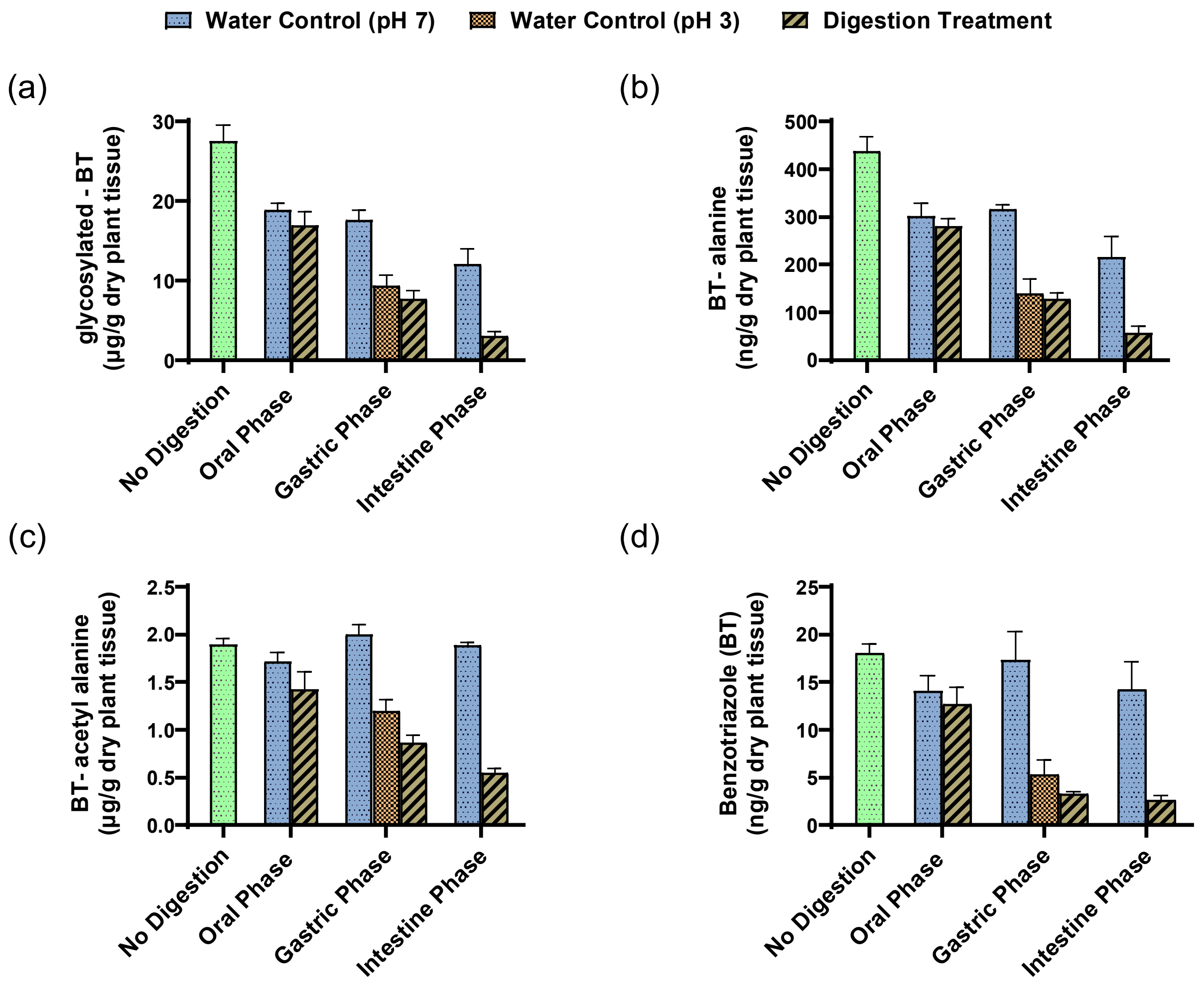

Figure S6: Mean mass (g/g freeze dry plant tissue) of (a) glycosylated-BT (b) BT-alanine (c) BT-acetyl alanine and (d) Benzotriazole in plant tissue extract for no digestion plants (n=4 boxes) and extracted after oral, gastric, and intestine phases for digestion treatment plants (n=4 boxes for each phase) and Water control plants (n=4 boxes for each phase); The error bars represent the standard error about the mean.

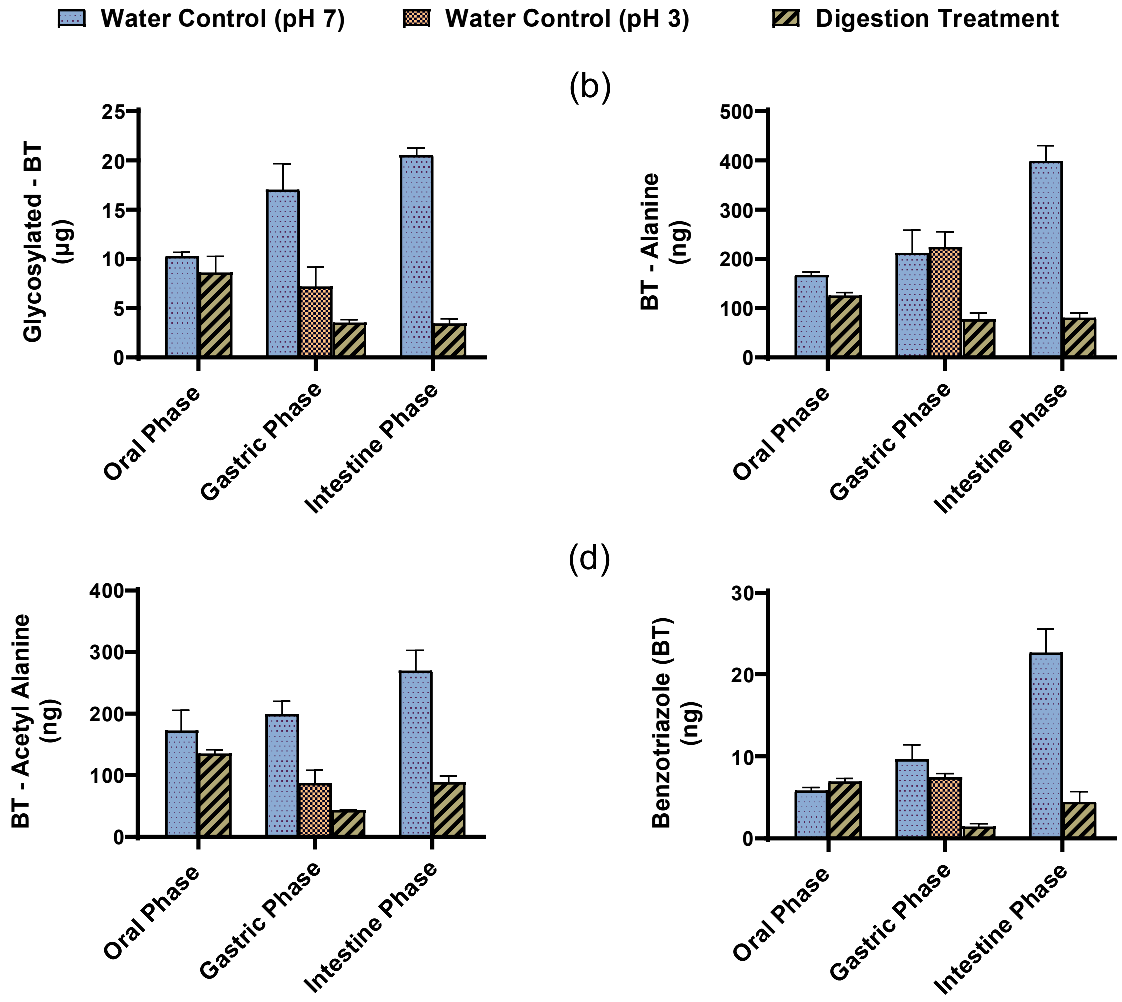

Figure S7: Mean mass of (a) glycosylated-BT (b) BT-alanine (c) BT-acetyl alanine and (d) Benzotriazole plant tissue digesta of digestion treatment plants (n=4 boxes for each phase) and water control plants (n=4 boxes for each phase).

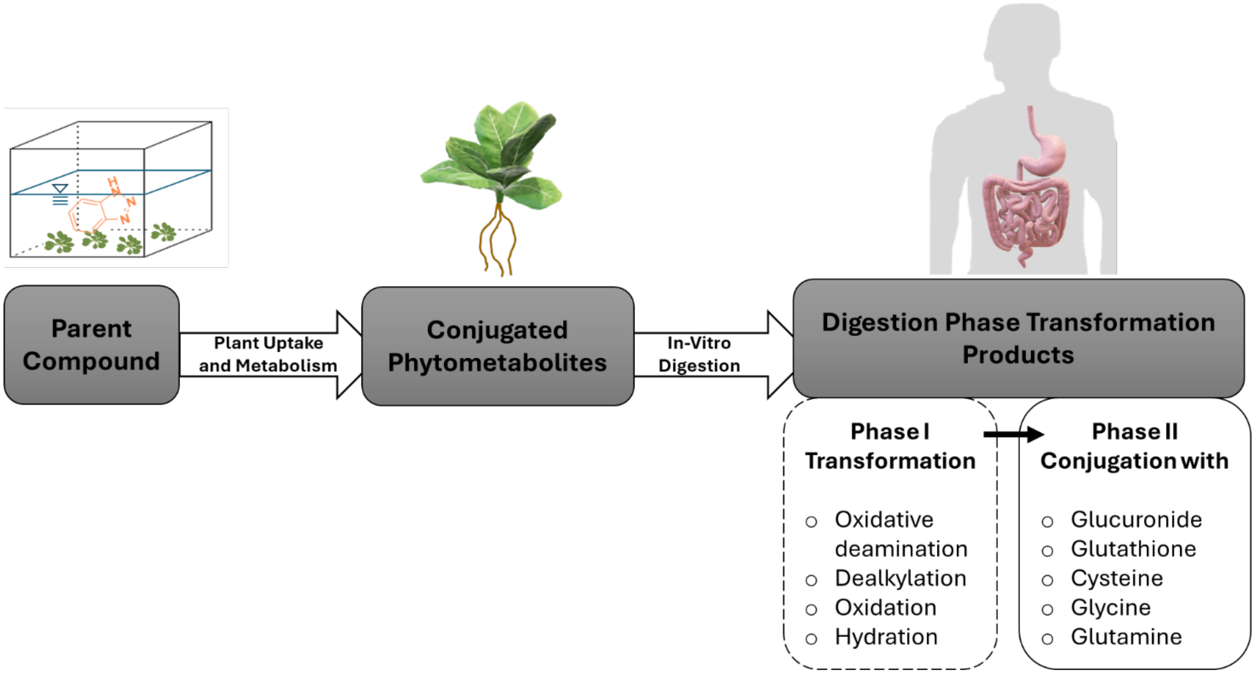

Figure S8: Flowchart showing the processes of parent benzotriazole transformation through plants and in-vitro digestion. The phases of digestion transformation are highlighted and correspond to the products shown in Figure 3 of the main text.

Table S9: Description of our use of the Schymanski framework[2], including study-specific sublevels

Confidence Level	Description	Data requirement	Data used for ***this study***
Level 1	*Confirmed structure* by reference standard	MS, MS^2, Retention Time, Reference Standard	MS, MS^2, Retention Time, Reference Standard
Level 2a	*Probable structure* by library spectrum match	MS, MS^2, Library MS^2	MS, MS^2, Library MS^2, Literature spectrum match (including our prior published work)[3]
Level 2b	*Probable structure* by diagnostic evidence	MS, MS^2, Experimental Data	MS, MS^2, Experimental Data, diagnostic MS^2 fragments, required fragment of parent compound benzotriazole; mass defect <10ppm
Level 3	*Tentative candidate* by structure, substituent, class	MS, MS^2, Experimental Data	MS, MS^2, Experimental Data, MS^2 fragment does not include parent compound but other fragments explained; mass defect <10ppm
Level 4	Unequivocal molecular formula	MS isotope/adduct	n/a
Level 5	Exact mass of interest	MS	n/a

BENZOTRIAZOLE PHYTOMETABOLITE DETAILS

Table S10:

*ppm error= (difference between measured m/z and exact mass of proposed ionized formula, in atomic mass units)/exact mass of proposed ionized formula) x10^6

Compound Name (spectra details included figure)	Proposed Metabolite Structure (Unionized)	Proposed Metabolite Formula (Unionized)	Confidence Level	Retention Time (min)	Measured m/z, Positive/ Negative Ion	Exact Mass of Proposed Ionized Formula	Accurate Mass Deviation (ppm)*	Fragment Measured m/z	Proposed Unionized Fragment Molecular Formula
glycosylated-BT (GBT) (Figure S7)	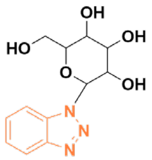	$C_{12}H_{15}N_3O_5$	<u>Level 1</u>: Confirmed structure by reference standard	1.371	282.10861 $[M+H]^{+1}$	282.10900	-1.38	120	$C_6H_6N_3$
Benzotriazole-alanine (BT-ala) (Figure S8)	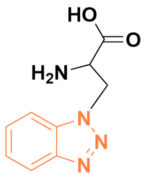	$C_9H_{10}N_4O_2$	<u>Level 1</u>: Confirmed structure by reference standard	1.121	207.08786 $[M+H]^{+1}$	207.08821	-1.69	161<br>134<br>132<br>120	$C_8H_9N_4$<br>$C_8H_8N_2$<br>$C_7H_8N_3$<br>$C_6H_6N_3$
Benzotriazole-acetyl alanine (BT-ac-ala) (Figure S9)	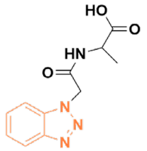	$C_{11}H_{12}N_4O_3$	<u>Level 1</u>: Confirmed structure by reference standard	3.089	247.08406 $[M-H]^{-1}$	247.08311	3.84	118	$C_6H_4N_3$
Benzotriazole-acetyl aspartic acid (BT-ac-ala-aspa) (Figure S10)	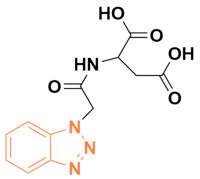	$C_{12}H_{12}N_4O_5$	<u>Level 2a:</u> Probable structure by library spectrum match	3.070	293.08817 $[M+H]^{+1}$	293.0886	-1.46	120<br>174	$C_6H_6N_3$<br>$C_6H_8NO_5$

DIGESTION PHASE TRANSFORMATION PRODUCT DETAILS

Table S11:

*Mass deviation (ppm) = (difference between measured m/z and exact mass of proposed ionized formula, in atomic mass units)/exact mass of proposed ionized formula) x10^6

								Fragments	
Compound Name (spectra details included figure)	Proposed Metabolite Structure (Unionized)	Proposed Metabolite Formula (Unionized)	Confidence Level	Retention Time (min)	Measured m/z, Positive/ Negative Ion	Exact Mass of Proposed Ionized Formula	Accurate Mass Deviation (ppm)*	Fragment Measured m/z	Proposed Unionized Fragment Molecular Formula
Glucuronide conjugated Glycosylated-BT (GBT-glc) (Figure S11)	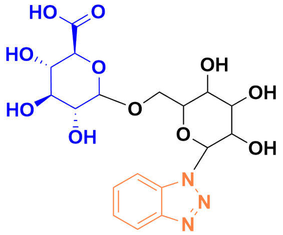	$C_{18}H_{23}N_3O_{11}$	Level 2b: Probable Structure by diagnostic evidence	3.916	456.12598 $[M+H]^{+1}$	456.12542	1.23	410.12112<br>367.07822<br>119.04869<br>90.05501	$C_{17}H_{22}N_3O_9$<br>$C_{15}H_{17}N_3O_8$<br>$C_6H_5N_3$<br>$C_3H_6O_3$
Glucuronide conjugated BT – Acetyl Alanine (BT-ac-ala-glc) (Figure S12)	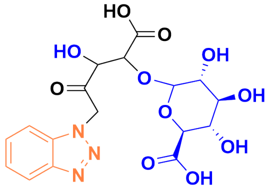	$C_{17}H_{19}N_3O_{11}$	Level 2b: Probable Structure by diagnostic evidence	3.300	442.11017 $[M+H]^{+1}$	442.10979	0.86	396.10367<br>118.04121	$C_{16}H_{18}N_3O_9$<br>$C_6H_4N_3$
Glucuronide conjugated BT- Alanine (BT-ala-glc) (Figure S13)	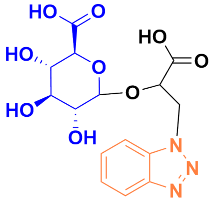	$C_{15}H_{17}N_3O_9$	Level 2b: Probable Structure by diagnostic evidence	2.484	384.10492 $[M+H]^{+1}$	384.10431	1.58	119.04917	$C_6H_5N_3$

								Fragments	
Compound Name (spectra details included figure)	Proposed Metabolite Structure (Unionized)	Proposed Metabolite Formula (Unionized)	Confidence Level	Retention Time (min)	Measured m/z, Positive/ Negative Ion	Exact Mass of Proposed Ionized Formula	Accurate Mass Deviation (ppm)*	Fragment Measured m/z	Proposed Unionized Fragment Molecular Formula
Glutathione conjugation of Glycosylated-BT (GBT-GSH) (Figure S14; *see also notes*)	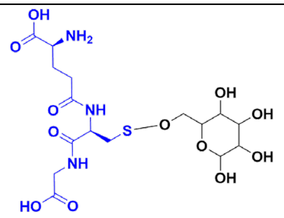	$C_{16}H_{27}N_3O_{12}S$	Level 3: Tentative candidate by structure, substituent, class	3.681	486.13675 $[M+H]^{+1}$	486.13937	-5.38	440.13389<br>131.09170<br>120.06554	$C_{15}H_{26}N_3O_{10}S$<br>$C_5H_{11}N_2O_2$<br>$C_4H_{10}NO_3$
Glutathione conjugated BT – Acetyl Alanine (BT-ac-ala-GSH) (Figure S15)	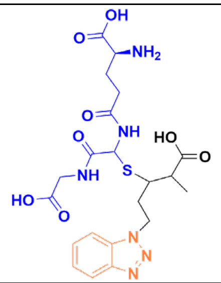	$C_{21}H_{28}N_6O_8S$	Level 3: Tentative candidate by structure, substituent, class	2.089	525.17670 $[M+H]^{+1}$	525.17713	-0.82	337.12161<br>319.11185	$C_{14}H_{19}N_5O_3S$<br>$C_{14}H_{17}N_5O_2S$
Cysteine conjugated BT – Acetyl Alanine (BT-ac-ala-cys) (Figure S16)	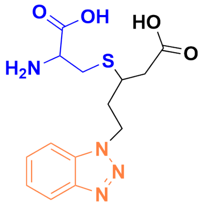	$C_{14}H_{18}N_4O_4S$	Level 3: Tentative candidate by structure, substituent, class	0.902	339.11227 $[M+H]^{+1}$	339.11270	-1.27	293.10672<br>276.08072<br>186.05835	$C_{13}H_{17}N_4O_2S$<br>$C_{13}H_{14}N_3O_2S$<br>$C_8H_{12}NO_2S$
Glycine conjugated BT – Acetyl Alanine (BT-ac-ala-gly) (Figure S17)	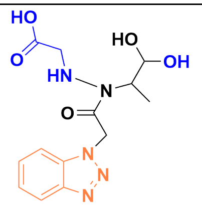	$C_{13}H_{17}N_5O_5$	Level 2b: Probable Structure by diagnostic evidence	7.488	346.11060 $[M + Na]^{+1}$	346.11274	-6.18	162.06760	$C_8H_8N_3O$

								Fragments	
Compound Name (spectra details included figure)	Proposed Metabolite Structure (Unionized)	Proposed Metabolite Formula (Unionized)	Confidence Level	Retention Time (min)	Measured m/z, Positive/ Negative Ion	Exact Mass of Proposed Ionized Formula	Accurate Mass Deviation (ppm)*	Fragment Measured m/z	Proposed Unionized Fragment Molecular Formula
Glycine conjugated BT – Alanine (BT-ala-gly) (Figure S18)	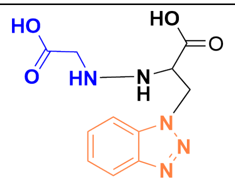	$C_{11}H_{13}N_5O_4$	Level 2b: Probable Structure by diagnostic evidence	5.529	302.08459 $[M + Na]^{+1}$	302.08652	-6.38	207.51744<br>119.04929	$C_9H_{11}N_4O_2$<br>$C_6H_5N_3$
Glutamine conjugated BT – Acetyl Alanine (BT-ac-ala-gln) (Figure S19)	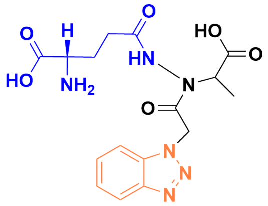	$C_{16}H_{20}N_6O_6$	Level 2b: Probable Structure by diagnostic evidence	7.677	413.11990 $[M + Na]^{+1}$	413.13420	4.95	223.05807<br>178.06238<br>118.04121	$C_{10}H_8N_4O$<br>$C_8H_8N_3O_2$<br>$C_6H_4N_3$
Glutamine conjugated BT – Alanine (BT-ala-gln) (Figure S20)	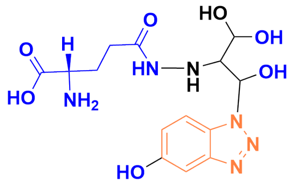	$C_{14}H_{20}N_6O_7$	Level 2b: Probable Structure by diagnostic evidence	4.754	407.12726 $[M + Na]^{+1}$	407.12912	-4.57	361.12146<br>320.09528<br>302.08517<br>166.03244<br>134.3554<br>105.04497	$C_{13}H_{18}N_6O_5Na$<br>$C_{11}H_{15}N_5O_5Na$<br>$C_{11}H_{13}N_5O_4$ Na<br>$C_5H_7N_2O_3Na$<br>$C_6H_4N_3O$<br>$C_6H_5N_2$
Glutamine conjugated Benzotriazole (BT-gln) (Figure S21)	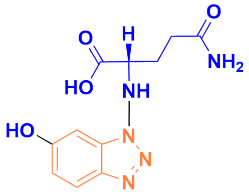	$C_{11}H_{13}N_5O_4$	Level 2b: Probable Structure by diagnostic evidence	5.815	302.08459 $[M + Na]^{+1}$	302.08652	-6.38	233.25186<br>207.05182<br>134.03544<br>118.04052	$C_{10}H_{12}N_5O_2$<br>$C_8H_7N_4O_3$<br>$C_6H_4N_3O$<br>$C_6H_4N_3$

DATA AVAILABILITY: In the event the HRMS data are of interest to any future research projects, the .mzML files used in this analysis have been deposited as a data set ("Metabolomic Transformation of Conjugated Benzotriazole Phytometabolites During In-vitro Digestion" DOI: 10.25820/data.007848). The .mzML file format was used to allow any future researchers the opportunity to conduct post-processing using proprietary or open-source tools of their choice, potentially to extend the data utility beyond the original study.

Table S12: pkCSM model predicted Adsorption, Distribution, Excretion and toxicity properties of the parent Benzotriazole, Benzotriazole phytometabolites and Digestion phase transformation products

**Compound name**	**Compound type**	**Conjugation with**	**Log P**	**Absorption property**			**Distribution properties**				**Excretion Property**	**Toxicity property**
				Water solubility (log mol/L)	Caco-permeability (log Papp in 10^{-6} cm/s)	Human intestinal absorption (% absorbed)	Volume of Distribution (Log L/kg)	Fraction Unbound	BBB permeability (log BB)	CNS Permeability (log PS)	Total clearance (log mL/min/kg)	Maximum Tolerated dose (log mg/kg/day)
Benzotriazole (BT)	Parent	-	0.96	-1.49	1.732	88.7	-0.02	0.56	-0.34	-2.21	0.04	0.04^{M}
Glycosylated BT (GBT)	Phytometabolites	Glucose	-2.34	-2.74	0.036	42.5	-0.74	0.24	-1.22	-4.34	0.11	0.93^{H}
BT-alanine (BT-ala)		alanine	-0.16	-1.90	-0.003	53.4	-0.52	0.36	-0.62	-3.10	0.36	0.95
BT-acetyl alanine (BT-ac-ala)		Acetyl alanine	0.02	-2.09	-0.065	62.3	-1.2	0.27	-0.77	-3.27	0.32	1.09^{H}

Compound name	Compound type	Conjugation with	Log P	Absorption properties			Distribution properties				Excretion property	Toxicity properties
				Water solubility (log mol/L)	Caco- 2 cells permeability (log Papp in 10^{-6} cm/s)	Human intestinal absorption (% absorbed)	Volume of Distribution (Log L/kg)	Fraction unbound	BBB[1] permeability (log BB)	CNS[2] Permeability (log PS)	Total clearance (log mL/min/kg)	Maximum Tolerated dose (log mg/kg/day)
Glucuronide conjugated GBT(GBT-Glc)	Digestion phase transformation products	Glucuronide	-3.68	-2.96	-0.186	0^{S}	-0.68	0.32	-1.80	-5.11	0.74	0.82^{H}
BT-ala-Glc			-2.21	-2.77	-0.498	0^{S}	-1.22	0.33	-1.67	-4.48	0.79	1.21^{H}
BT-ac-ala-Glc			-3.28	-2.88	-0.3	0^{S}	-0.88	0.36	-1.74	-5.34	1.37	1.37^{H}
Glyc-GSH		Glutathione	-4.68	-2.84	-0.901	0^{S}	-0.54	0.54	-2.64	-5.70	-0.09	0.46^{H}

BT-ac-ala (BT-ac-ala-GSH)	Digestion phase transformation products		-0.52	-2.89	-0.68	0	-0.11	0.43	-2.40	-4.61	-0.54	0.57[H]
BT-ac-ala-cys		Cysteine	-0.81	-2.89	-0.185	26.0[S]	-0.32	0.44	-1.46	-3.79	0.13	0.55[H]
BT-ala-gly		Glycine	-1.71	-2.64	-0.198	35.90[S]	-0.86	0.38	-1.20	-4.25	0.71	0.95[H]
BT-ac-ala-gly			-0.68	-2.55	-0.357	51.13	-2.139	0.26	-1.71	-4.52	0.54	1.42[H]
BT-ala-gln		Glutamine	-2.88	-2.89	-0.564	0[S]	-0.21	0.491	-2.378	-5.171	0.23	0.65[H]
BT-ac-ala-gln			-1.04	-2.89	-0.598	20.24	-0.22	0.48	-1.81	-4.51	-0.38	0.73[H]
BT-gln			-0.60	-2.67	-0.192	31.73[S]	-0.80	0.28	-1.21	-4.05	0.84	1.33[H]

[1]Blood-Brain-Barrier, [2]Central Nervous System, [S] P-glycoprotein substrate, [M] Mutagenic, [H] Hepatotoxic

Mass Spectra and Structures Details of Benzotriazole Phytometabolites

Glycosylated- Benzotriazole

MS:

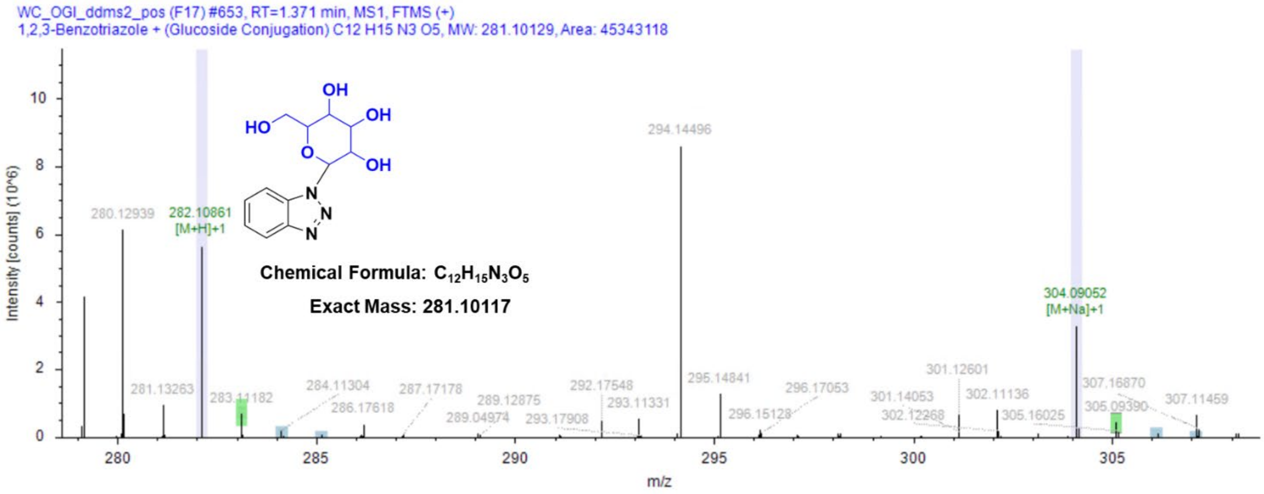

MS^2:

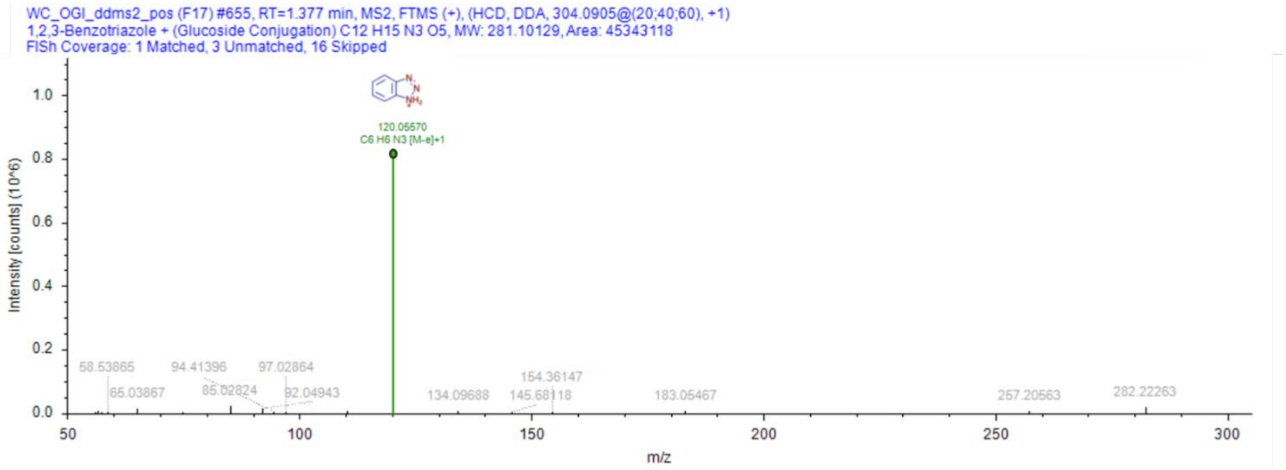

Figure S9: MS and MS^2 spectra of Glycosylated Benzotriazole phytometabolite

Benzotriazole-alanine

MS:

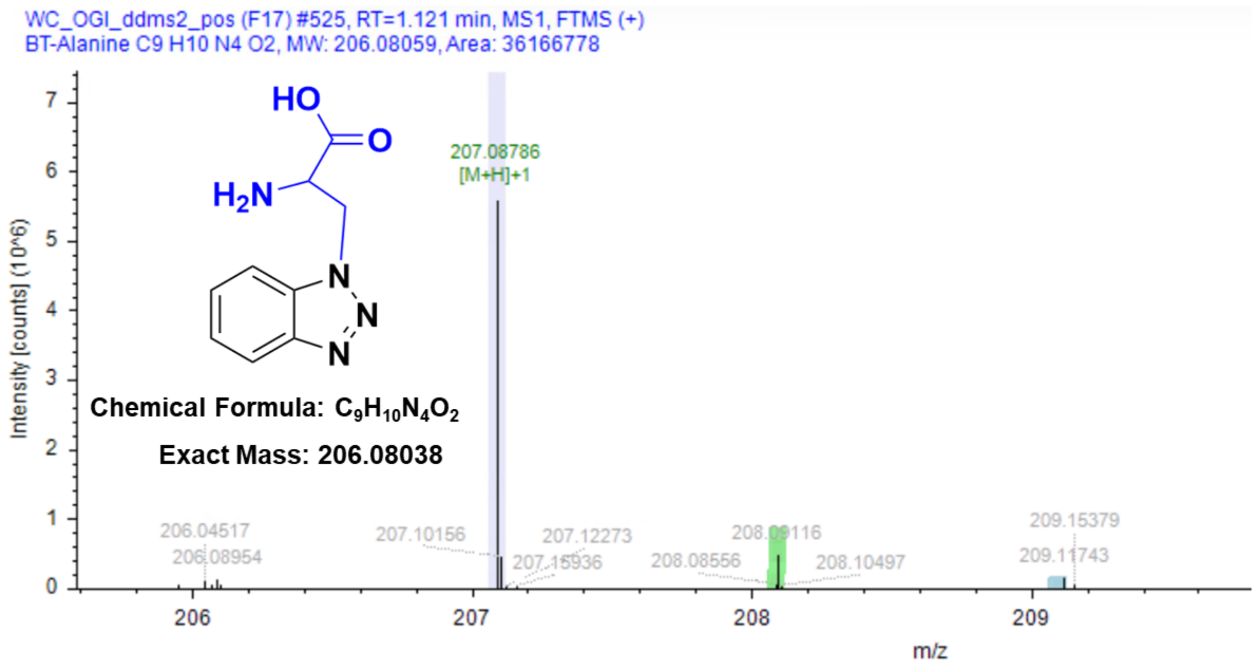

MS^2:

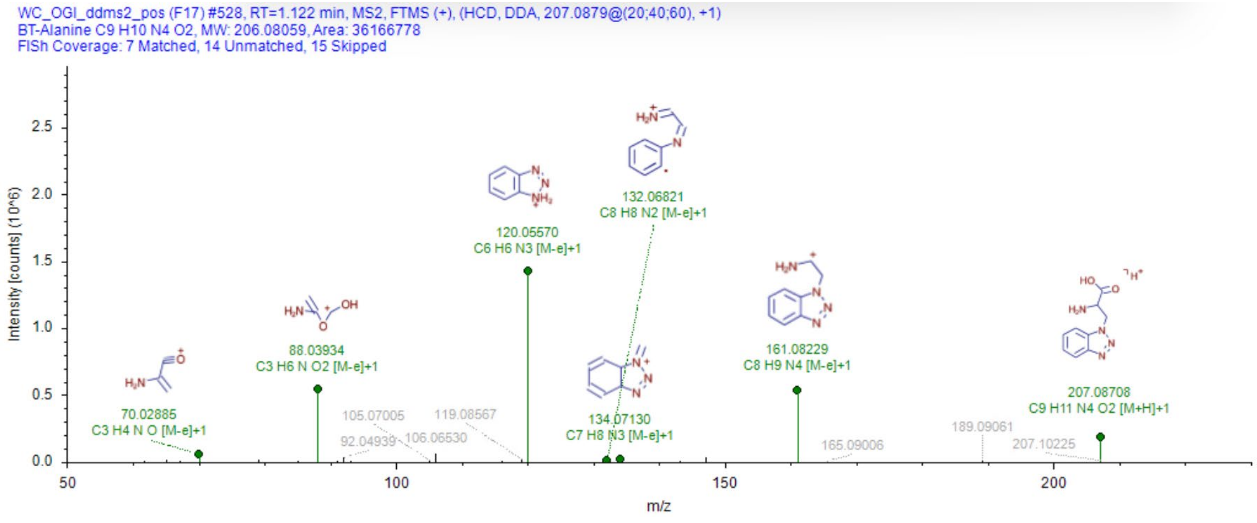

Figure S10: MS and MS^2 spectra of Benzotriazole-alanine phytometabolite

Benzotriazole-acetyl alanine

MS:

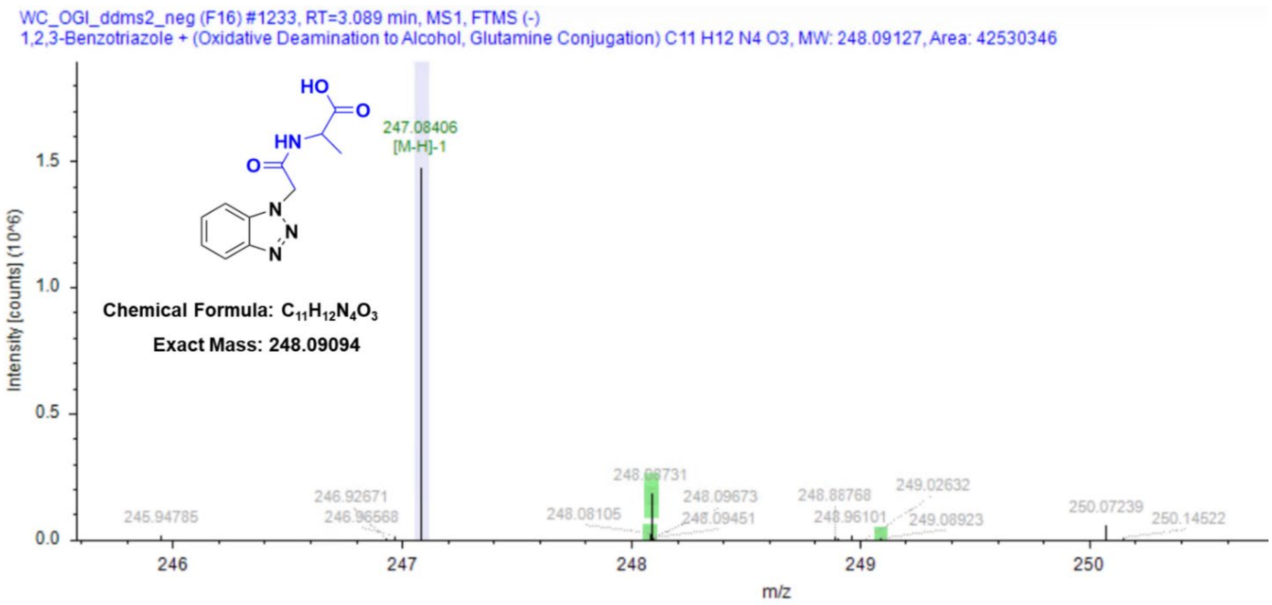

MS^2:

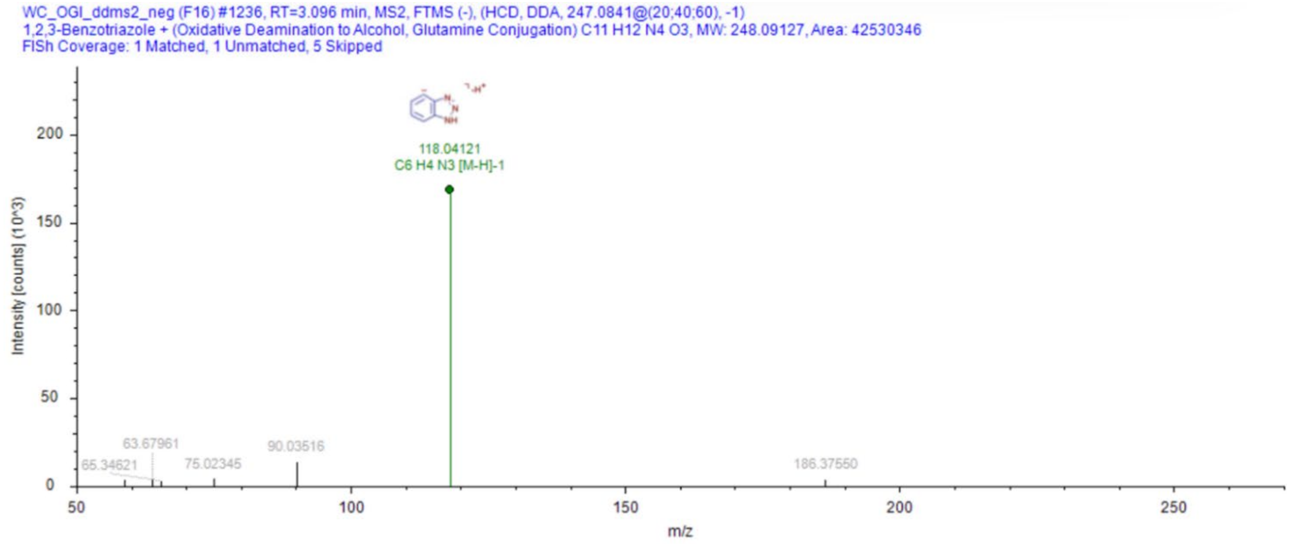

Figure S11: MS and MS^2 spectra of Benzotriazole acetyl alanine phytometabolite

Benzotriazole-acetyl aspartic acid

MS:

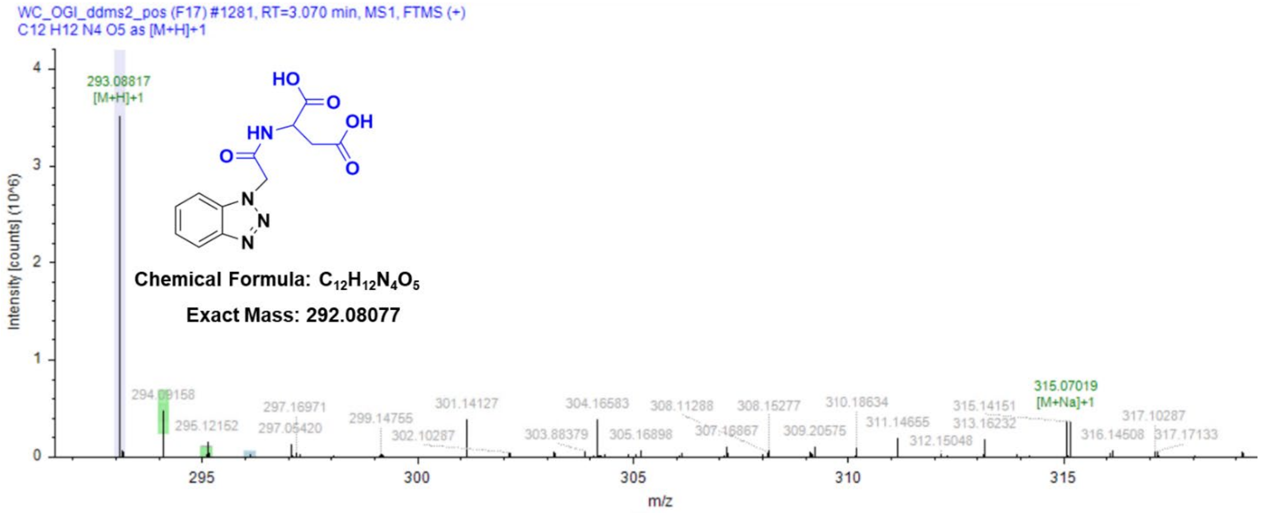

MS^2:

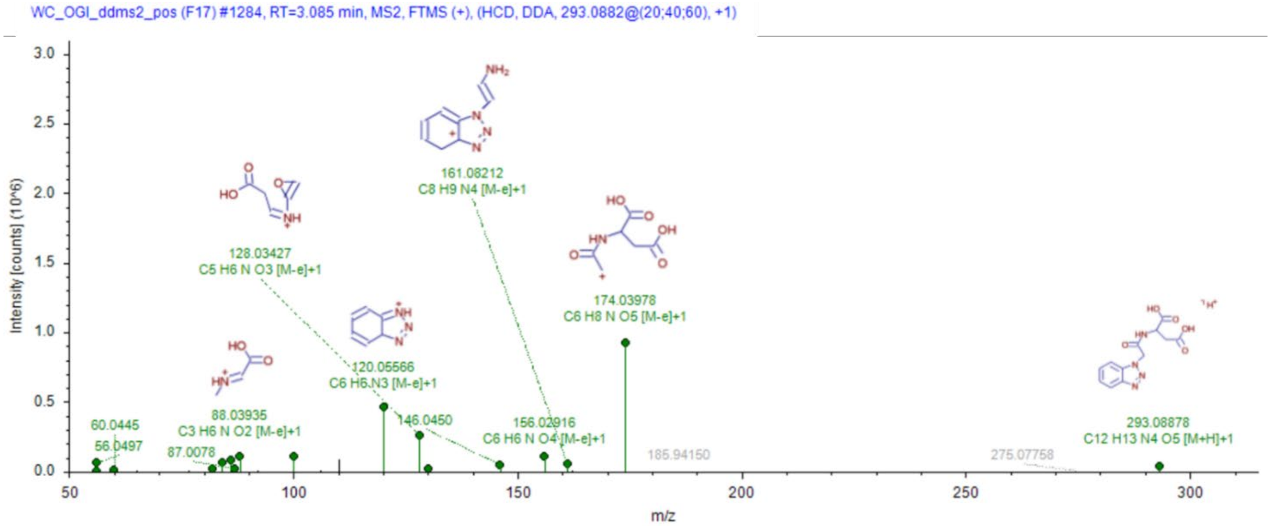

Figure S12: MS and MS^2 spectra of Benzotriazole acetyl aspartic acid phytometabolite

Mass Spectra and Structures details of Digestion Phase Transformation Products

Products with Glucuronide Conjugation

For Glycosylated benzotriazole

MS:

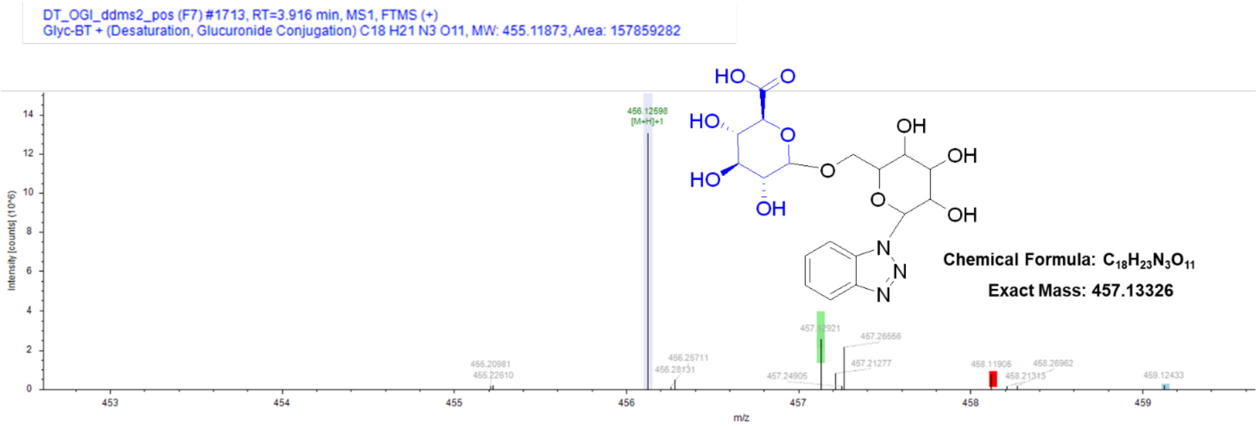

MS^2:

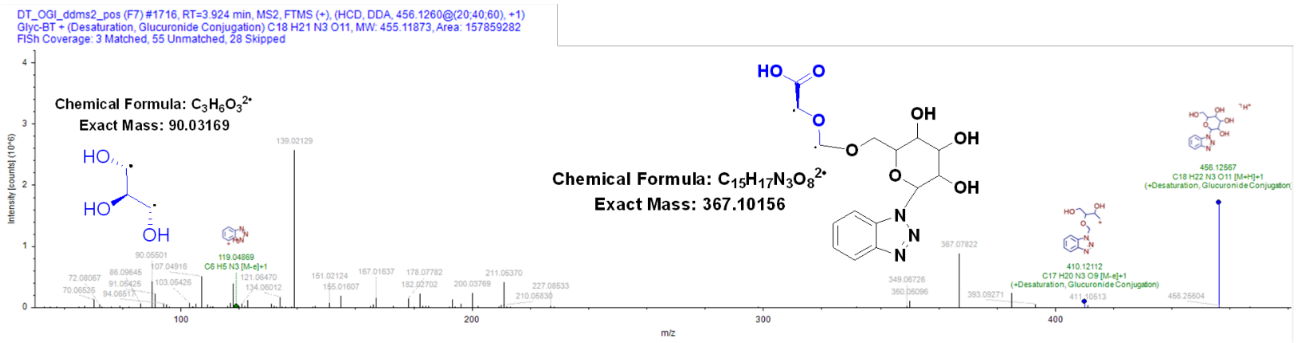

Figure S13: MS and MS^2 spectra of Glucuronide conjugated Glycosylated benzotriazole

For Benzotriazole-acetyl alanine

MS:

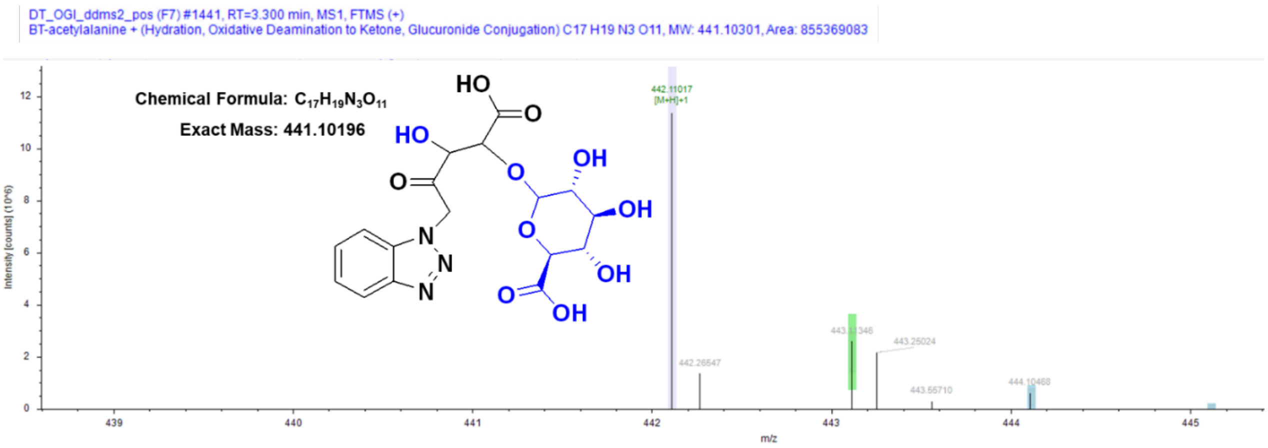

MS^2:

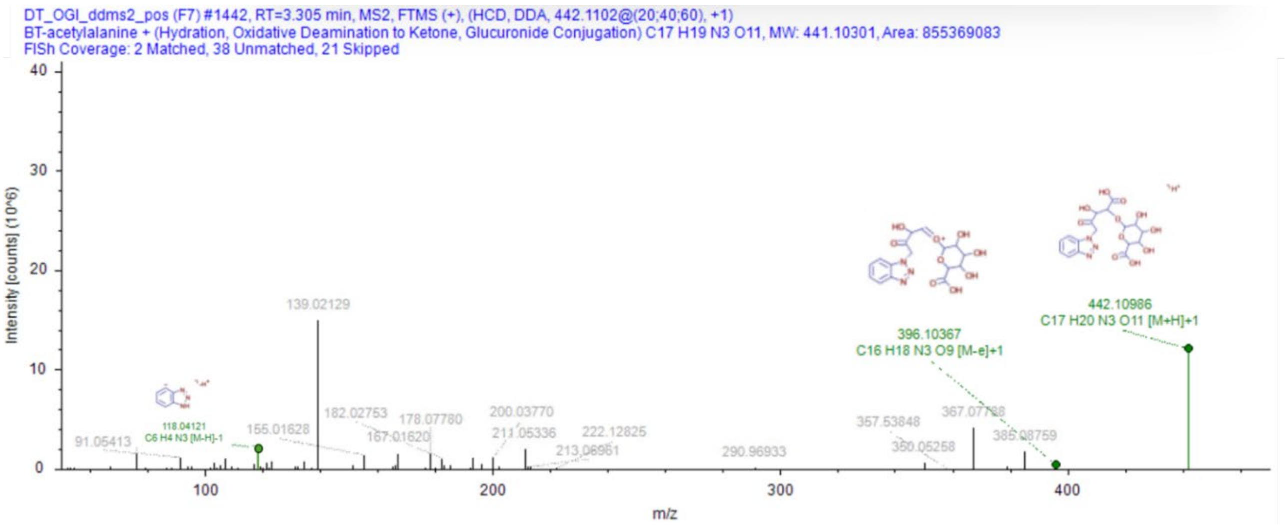

([Link to Summary Table](#))

Figure S14: MS and MS^2 spectra of Glucuronide conjugated Benzotriazole-acetyl alanine

For Benzotriazole-alanine

MS:

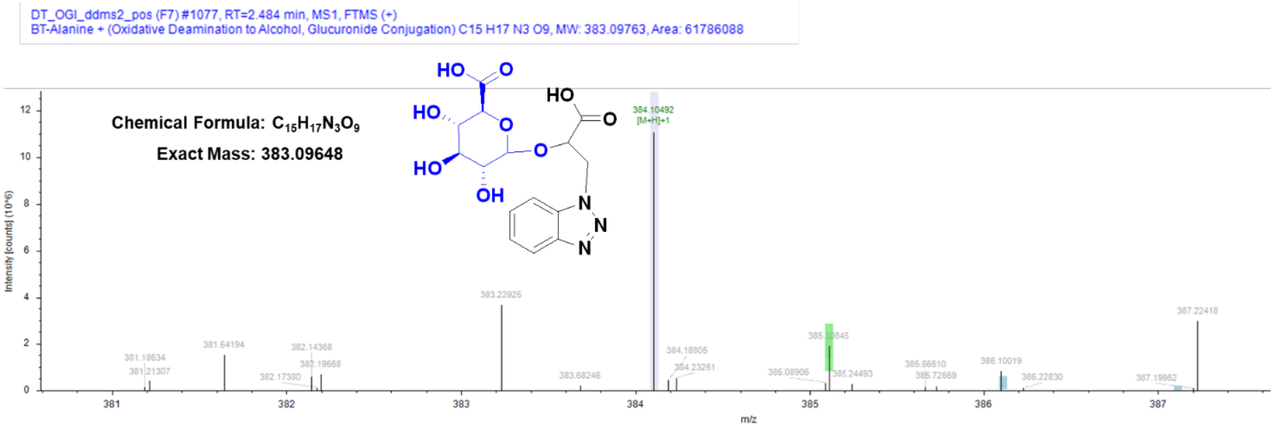

MS^2:

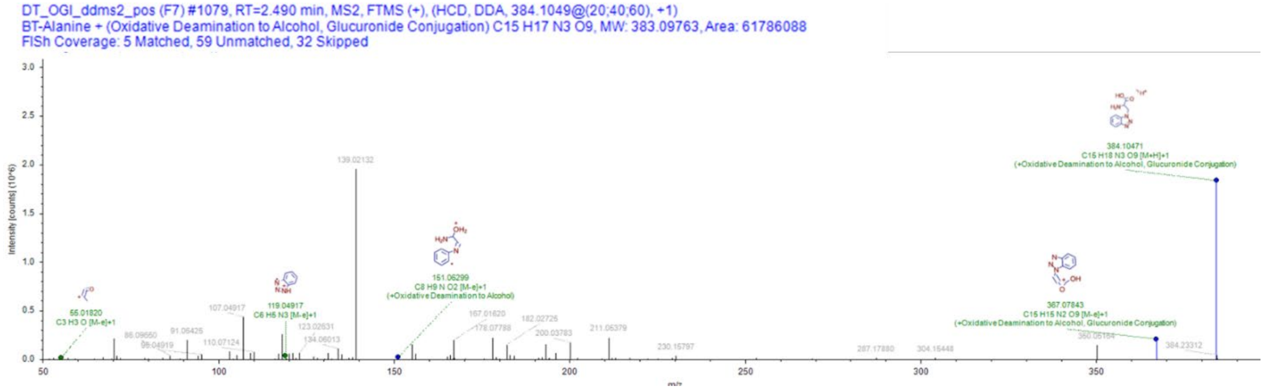

(Link to Summary Table)

Figure S15: MS and MS^2 spectra of Glucuronide conjugated Benzotriazole-alanine

Products with Glutathione Conjugation

For Glycosylated Benzotriazole

MS:

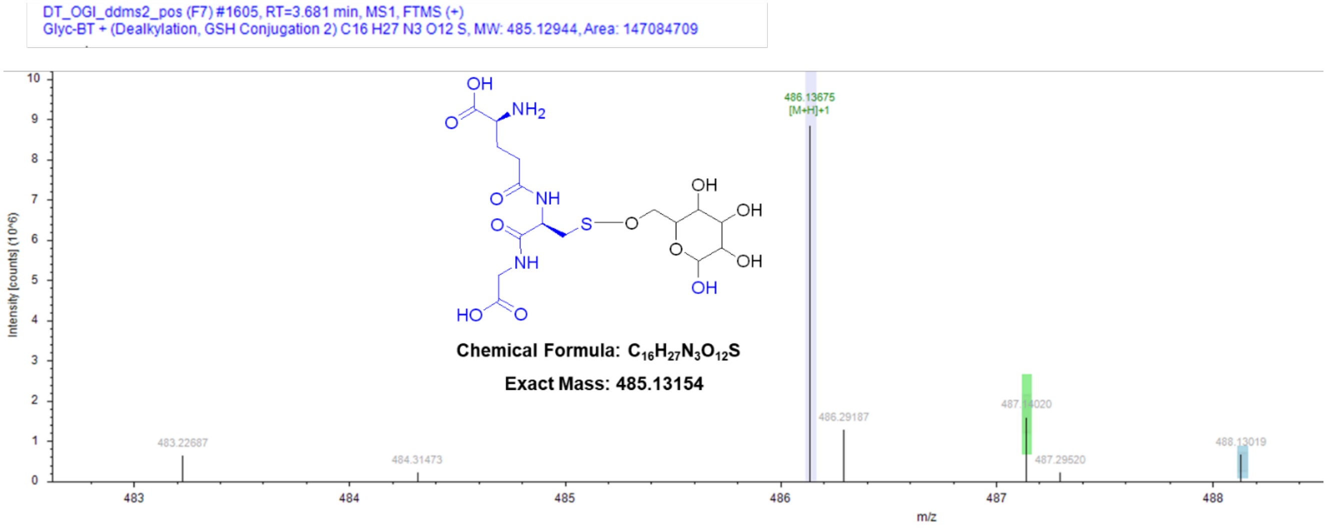

MS^2:

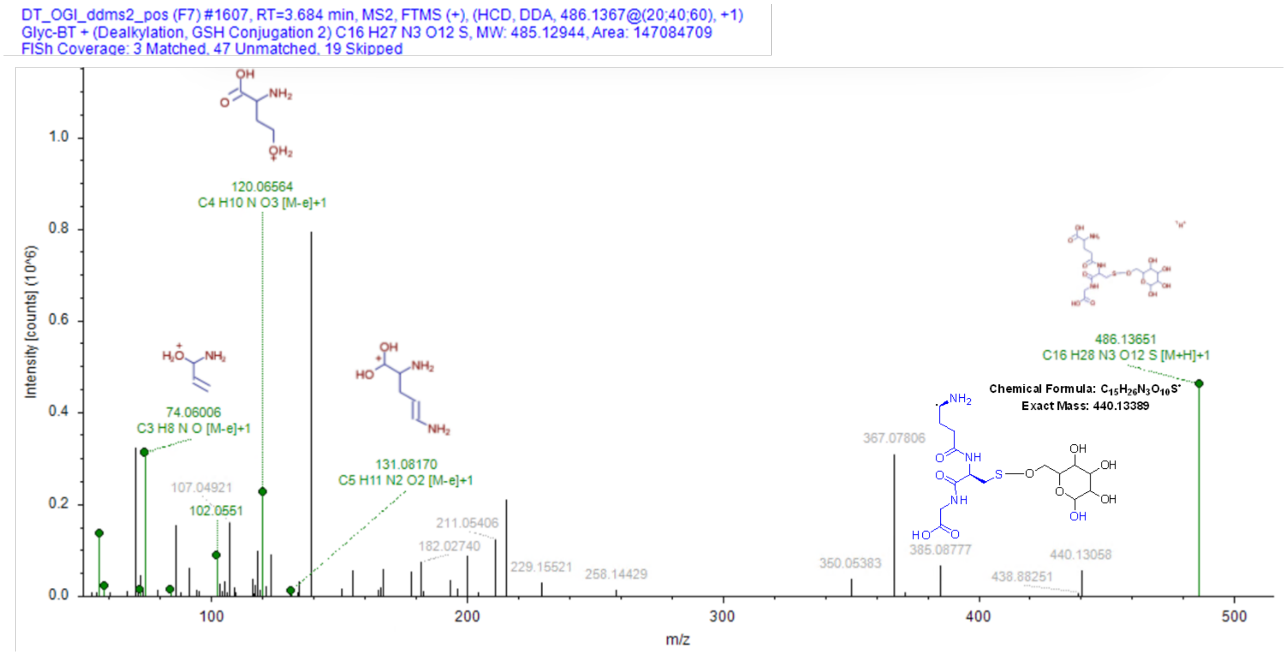

(Link to Summary Table)

MS of benzotriazole attached glutathione conjugates of glycosylated-Benzotriazole:

NOTE: We also found the benzotriazole attached glutathione conjugates of glycosylated-Benzotriazole(glyc-BT)[pictured below, Figure S14] as an expected transformation product of glyc-BT by searching for the exact mass of interest. Nevertheless, the compound was not found using the initial methodological screening because the peak area was $<10^8$, there was no MS2, and FISh coverage was zero; thus, the BT-conjugated form is not included in Figure 3 of the manuscript for the sake of methodological consistency but is highly likely a transformation product.

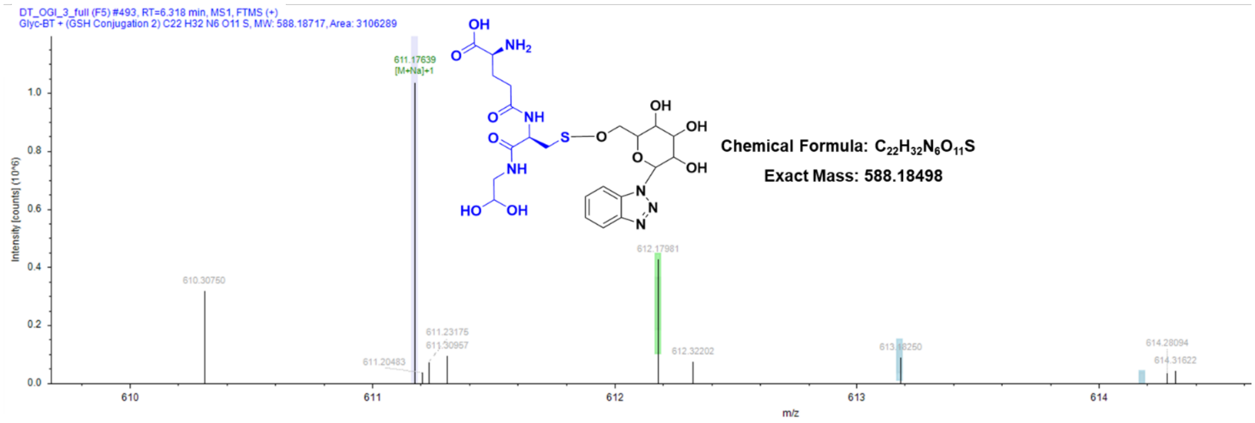

Figure S16: MS and MS^2 spectra of Glutathione conjugated transformation product of glycosylated-Benzotriazole.

For Benzotriazole-acetyl alanine

MS:

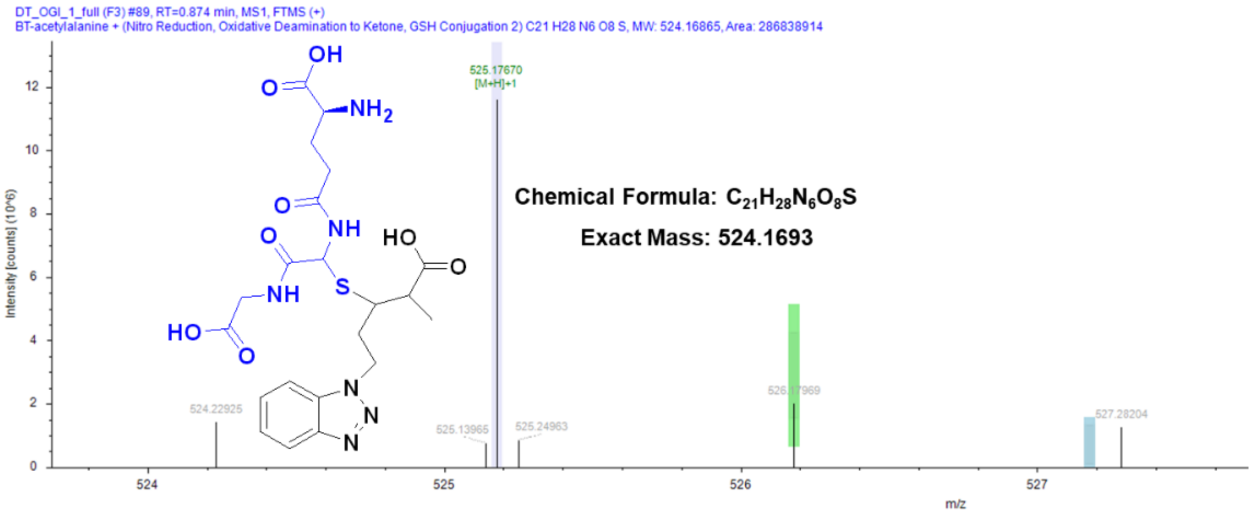

MS^2:

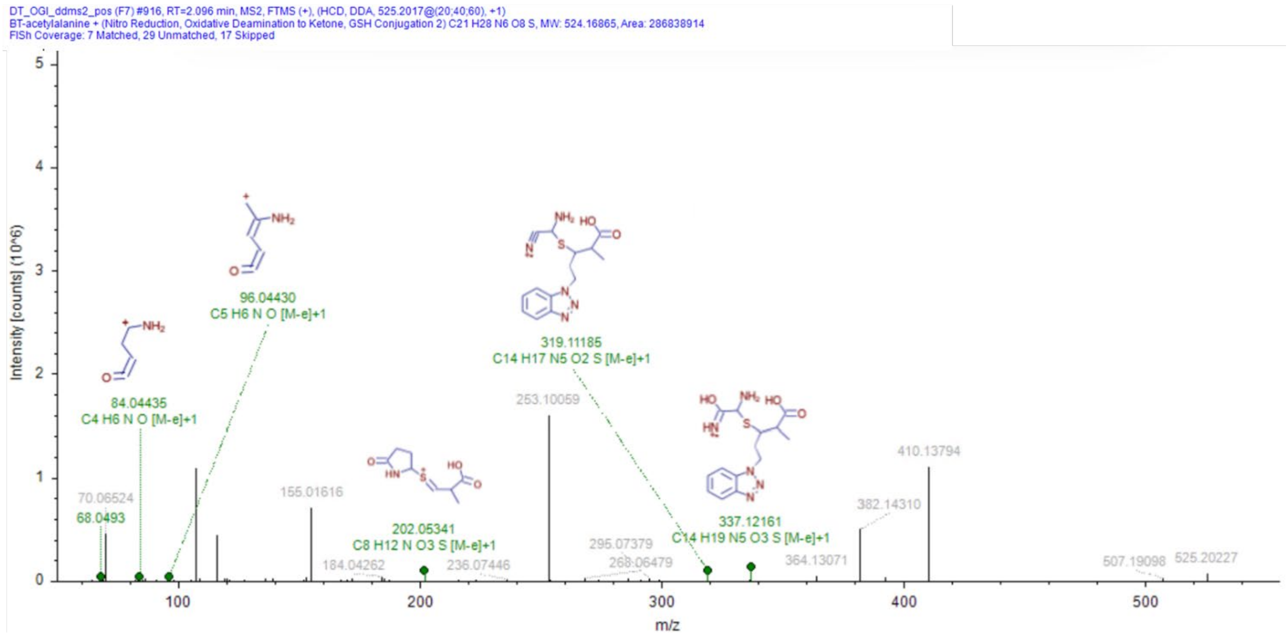

(Link to Summary Table)

Figure S17: MS and MS^2 spectra of Glutathione conjugated Benzotriazole-acetyl alanine

Product with Cysteine Conjugation

For Benzotriazole-acetyl alanine

MS:

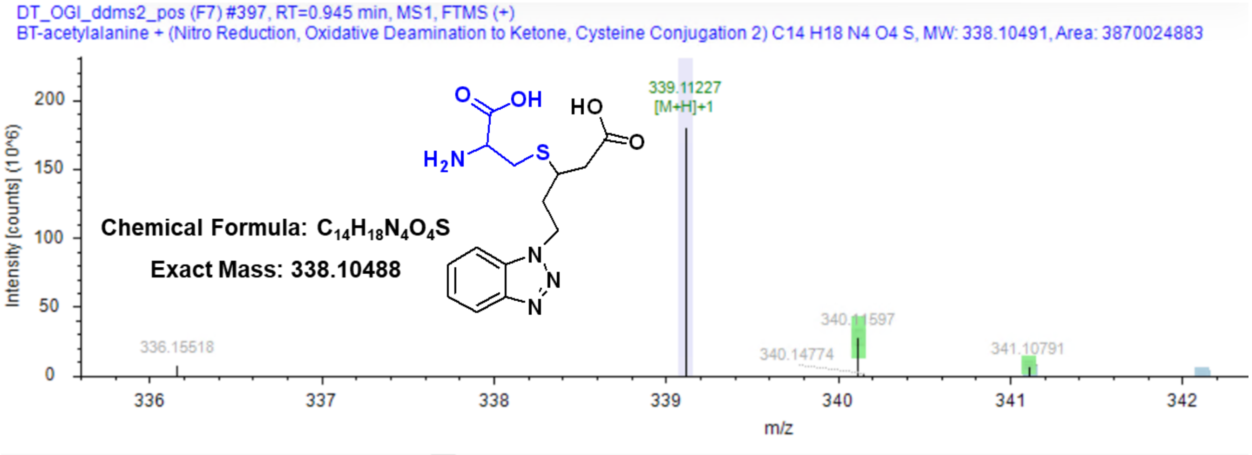

MS^2:

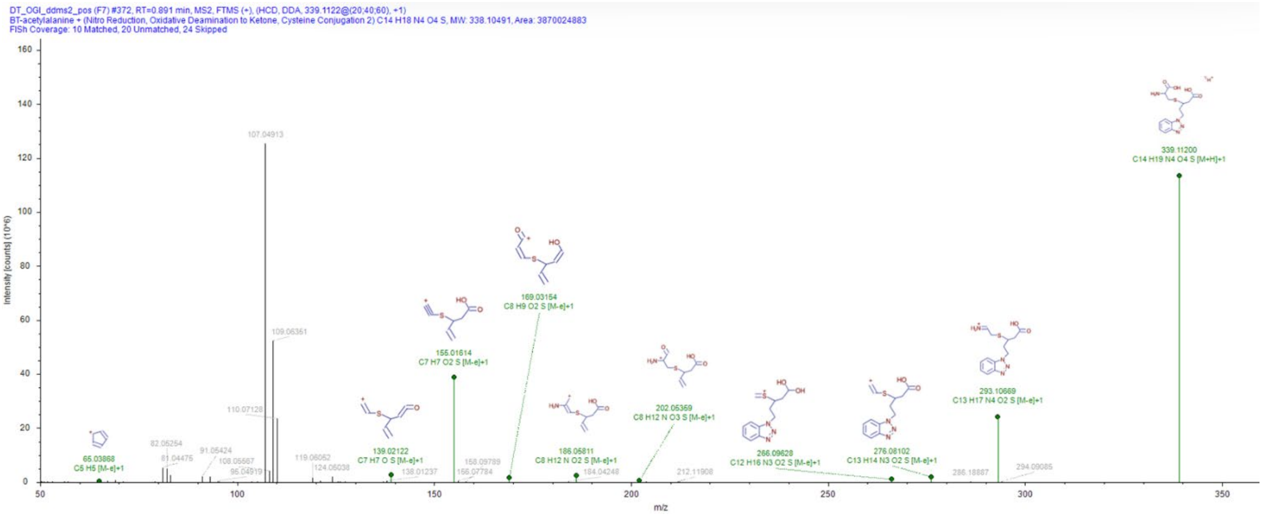

(Link to Summary Table)

Figure S18: MS and MS^2 spectra of Cysteine conjugated Benzotriazole acetyl alanine

Products with Glycine Conjugation

<u>For Benzotriazole-acetyl alanine</u>

MS:

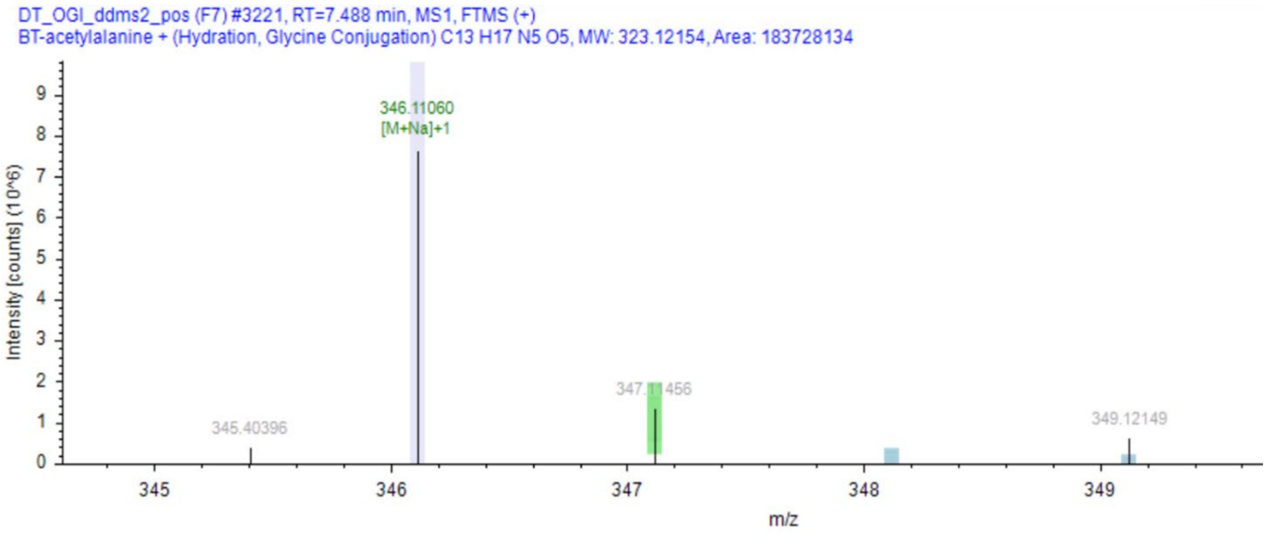

MS^2:

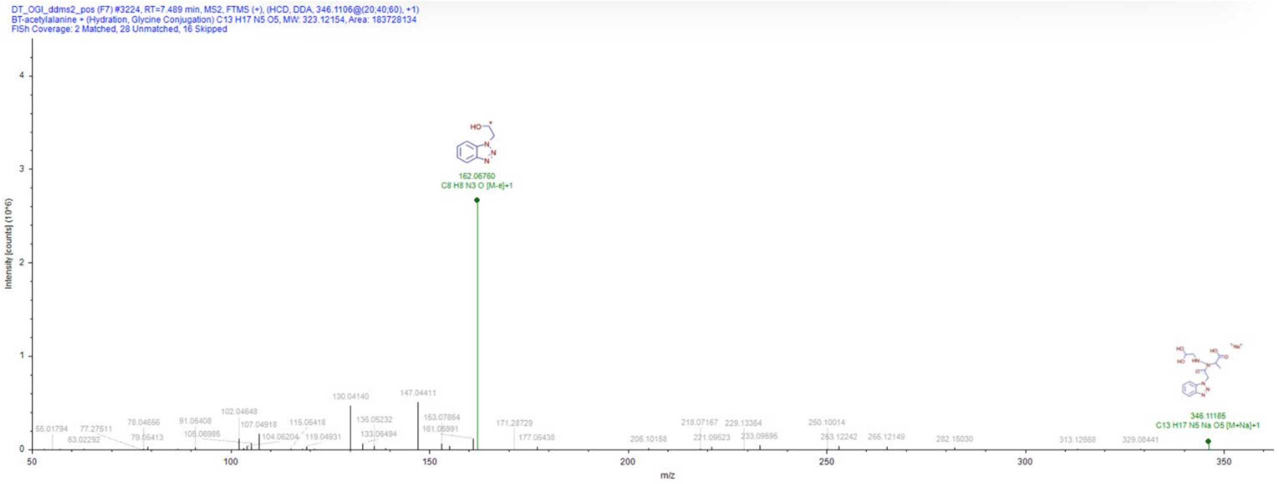

(Link to Summary Table)

Figure S19: MS and MS^2 spectra of Glycine conjugated Benzotriazole acetyl alanine

For Benzotriazole-alanine

MS:

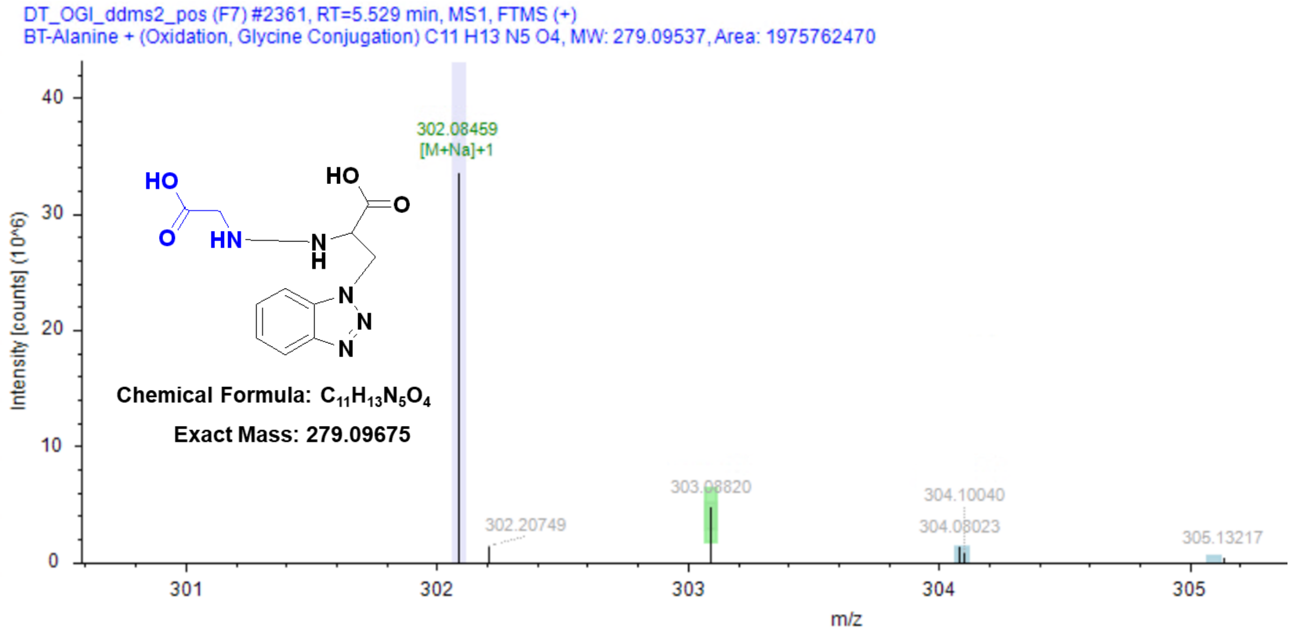

MS^2:

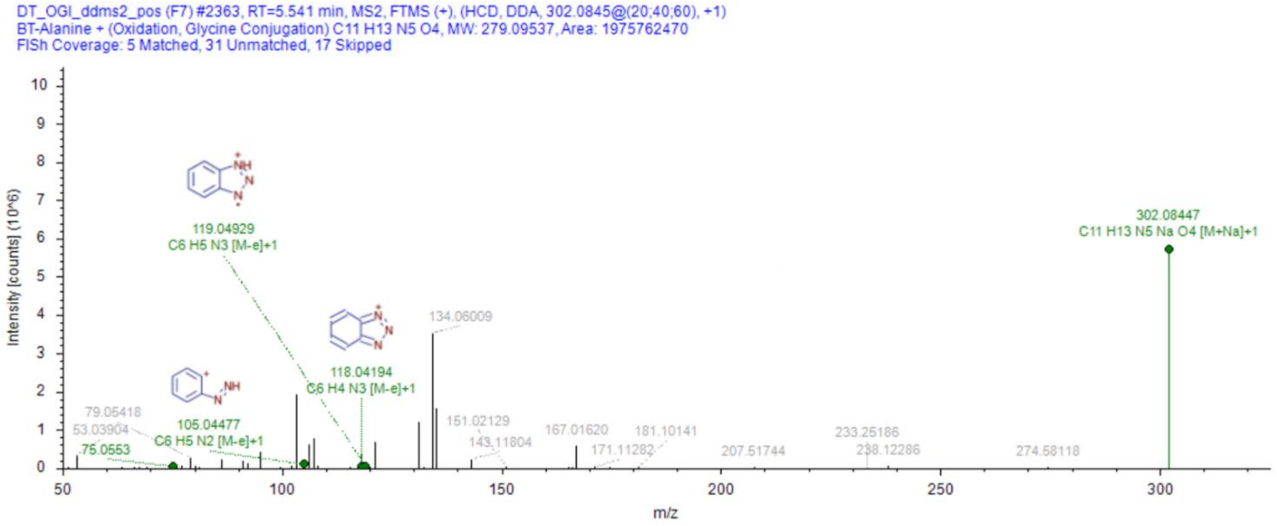

(Link to Summary Table)

Figure S20: MS and MS^2 spectra of Glycine conjugated Benzotriazole alanine

Products with Glutamine Conjugation

<u>For Benzotriazole- acetyl alanine</u>

MS:

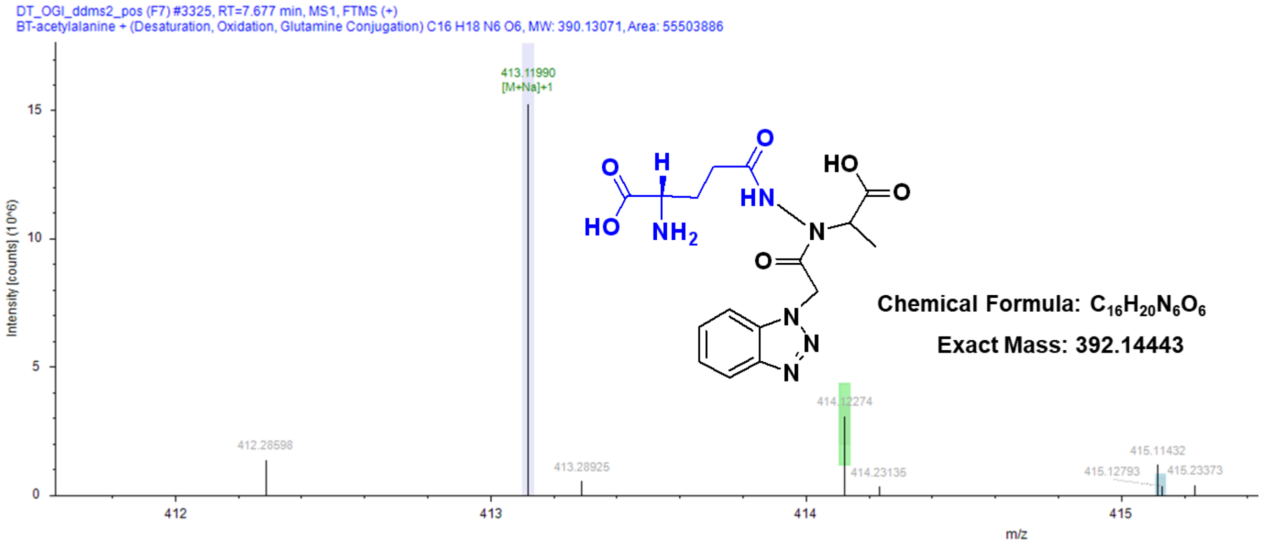

MS^2:

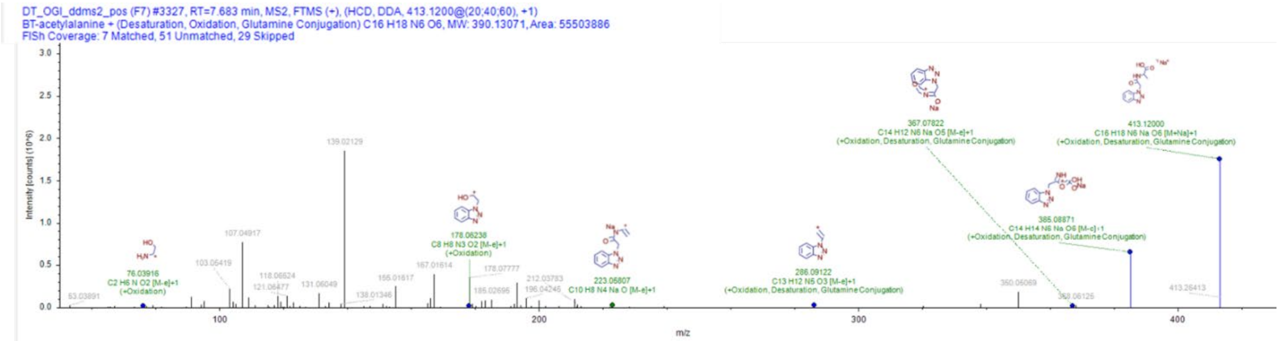

(<u>Link to Summary Table</u>)

Figure S21: MS and MS^2 spectra of Glutamine conjugated Benzotriazole acetyl alanine

For Benzotriazole-alanine

MS:

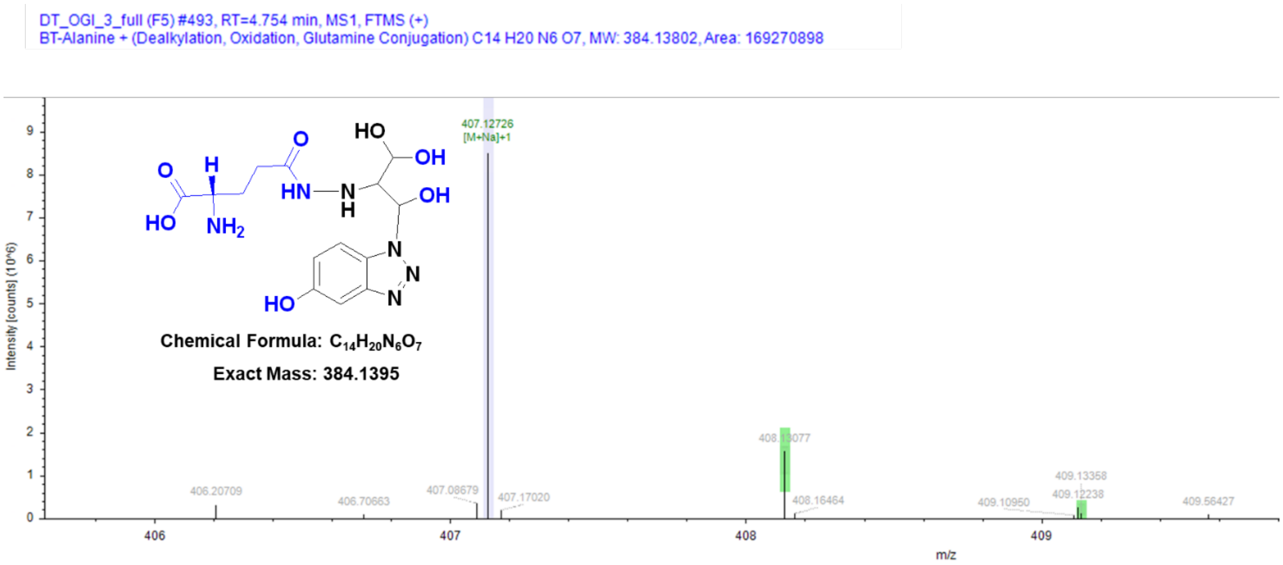

MS^2:

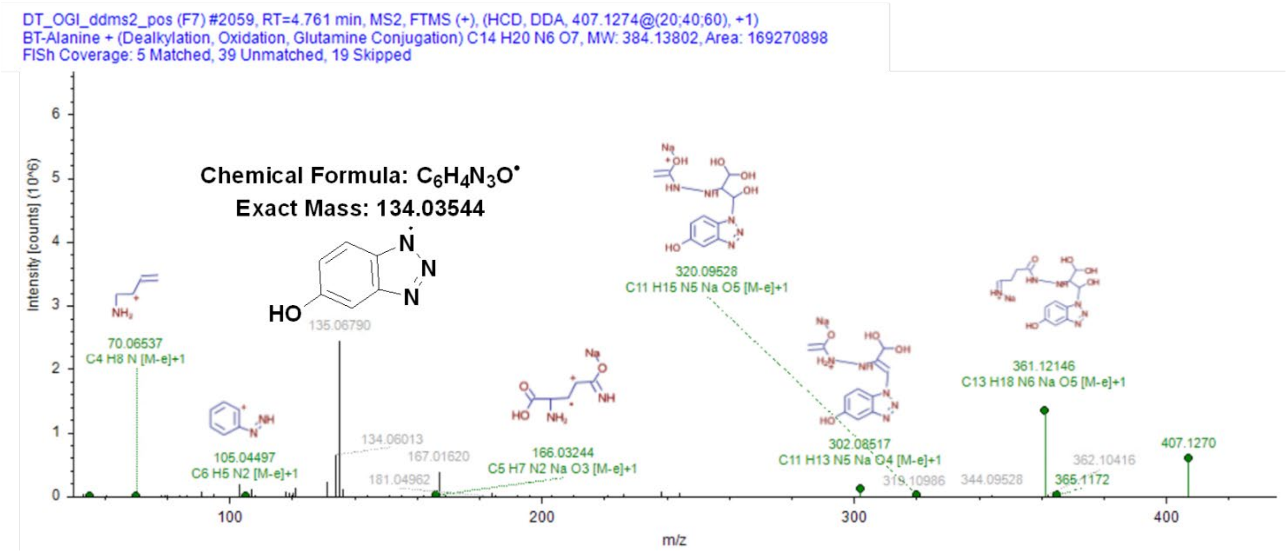

(Link to Summary Table)

Figure S22: MS and MS^2 spectra of Glutamine conjugated Benzotriazole alanine

For Benzotriazole

MS:

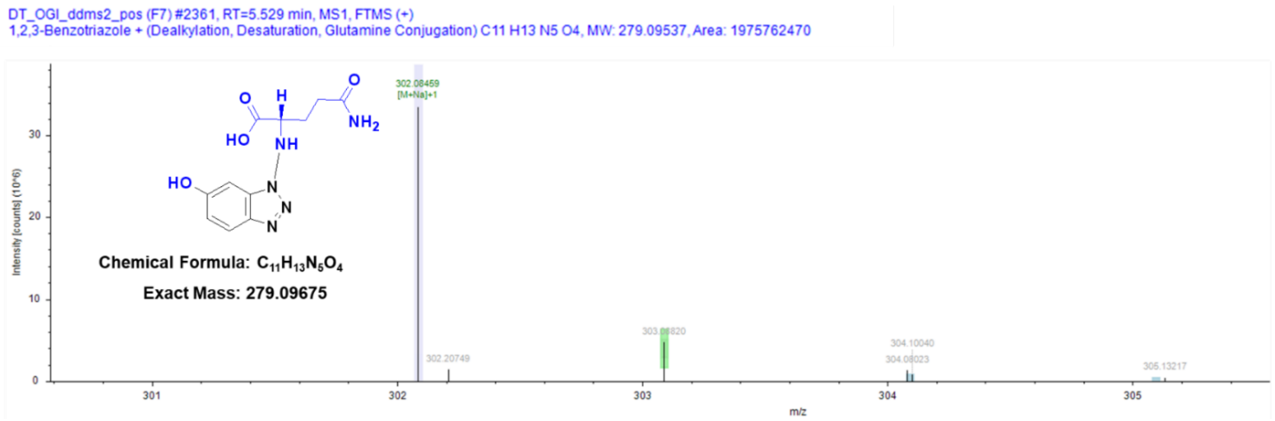

MS^2:

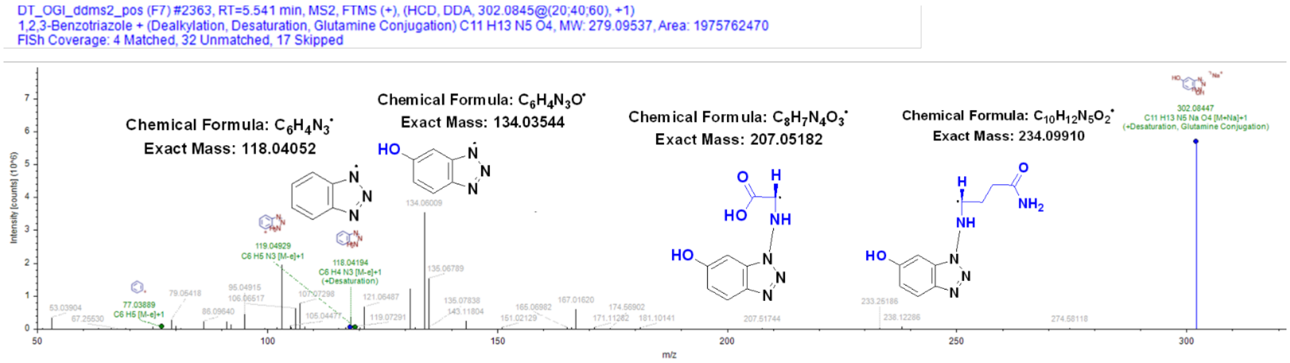

(Link to Summary Table)

Figure S23: MS and MS^2 spectra of Glutamine conjugated Benzotriazole

REFERENCES

(1) Brodkorb, A.; Egger, L.; Alminger, M.; Alvito, P.; Assunção, R.; Ballance, S.; Bohn, T.; Bourlieu-Lacanal, C.; Boutrou, R.; Carrière, F.; Clemente, A.; Corredig, M.; Dupont, D.; Dufour, C.; Edwards, C.; Golding, M.; Karakaya, S.; Kirkhus, B.; Le Feunteun, S.; Lesmes, U.; Macierzanka, A.; Mackie, A. R.; Martins, C.; Marze, S.; McClements, D. J.; Ménard, O.; Minekus, M.; Portmann, R.; Santos, C. N.; Souchon, I.; Singh, R. P.; Vegarud, G. E.; Wickham, M. S. J.; Weitschies, W.; Recio, I. INFOGEST Static in Vitro Simulation of Gastrointestinal Food Digestion. *Nat. Protoc.* **2019**, *14* (4), 991–1014. https://doi.org/10.1038/s41596-018-0119-1.

(2) Schymanski, E. L.; Jeon, J.; Gulde, R.; Fenner, K.; Ruff, M.; Singer, H. P.; Hollender, J. Identifying Small Molecules via High Resolution Mass Spectrometry: Communicating Confidence. *Environ. Sci. Technol.* **2014**, *48* (4), 2097–2098. https://doi.org/10.1021/es5002105.

(3) LeFevre, G. H.; Müller, C. E.; Li, R. J.; Luthy, R. G.; Sattely, E. S. Rapid Phytotransformation of Benzotriazole Generates Synthetic Tryptophan and Auxin Analogs in Arabidopsis. *Environ. Sci. Technol.* **2015**, *49* (18), 10959–10968. https://doi.org/10.1021/acs.est.5b02749.
